# Neural Speech Tracking during Selective Attention: A Spatially Realistic Audiovisual Study

**DOI:** 10.1523/ENEURO.0132-24.2025

**Published:** 2025-06-18

**Authors:** Paz Har-shai Yahav, Eshed Rabinovitch, Adi Korisky, Renana Vaknin Harel, Martin Bliechner, Elana Zion Golumbic

**Affiliations:** ^1^The Gonda Center for Multidisciplinary Brain Research, Bar Ilan University, Ramat Gan, 5290002, Israel; ^2^Department of Psychology, Carl von Ossietzky Universität Oldenburg, Germany

**Keywords:** EEG, selective attention, spatial, speech processing, TRF

## Abstract

Paying attention to a target talker in multitalker scenarios is associated with its more accurate neural tracking relative to competing non-target speech. This “neural bias” to target speech has largely been demonstrated in experimental setups where target and non-target speech are acoustically controlled and interchangeable. However, in real-life situations this is rarely the case. For example, listeners often look at the talker they are paying attention to while non-target speech is heard (but not seen) from peripheral locations. To enhance the ecological-relevance of attention research, here we studied whether neural bias toward target speech is observed in a spatially realistic audiovisual context and how this is affected by switching the identity of the target talker. Group-level results show robust neural bias toward target speech, an effect that persisted and generalized after switching the identity of the target talker. In line with previous studies, this supports the utility of the speech-tracking approach for studying speech processing and attention in spatially realistic settings. However, a more nuanced picture emerges when inspecting data of individual participants. Although reliable neural speech tracking could be established in most participants, this was not correlated with neural bias or with behavioral performance, and >50% of participants showed similarly robust neural tracking of both target and non-target speech. These results indicate that neural bias toward the target is not a ubiquitous, or necessary, marker of selective attention (at least as measured from scalp-EEG), and suggest that individuals diverge in their internal prioritization among concurrent speech, perhaps reflecting different listening strategies or capabilities under realistic conditions.

## Significance Statement

This work contributes to ongoing efforts to study the neural mechanisms involved in selective attention to speech under ecologically relevant conditions, emulating the type of speech materials, multisensory experience, and spatial realism of natural environments. Group-level results show that under these more realistic conditions, the hallmark signature of selective attention—namely, the modulation of sensory representation and its robustness to switches in target identity—is conserved, at least at the group level. At the same time, results point to an underlying diversity among participants in how that this modulation manifests, raising the possibility that differences in listening strategies, motivation, or personal traits lead to differences in the way that individuals encode and process competing stimuli, under ecological conditions.

## Introduction

Effectively directing attention to a particular talker, and prioritizing its processing over competing non-target speech, can be challenging. Selective attention to speech has been associated at the neural level with enhanced neural tracking of target speech, compared with non-target speech. This “neural bias” for target speech has been demonstrated in numerous EEG and MEG studies of selective attention to speech (Kerlin et al., 2010; Ding et al., 2012; Zion Golumbic, et al., 2013b; O’Sullivan et al., 2015; Fiedler et al., 2019) and mirrors similar effects of selective attention in modulating sensory responses to simpler stimuli (Broadbent, 1958; Treisman, 1969; Hillyard et al., 1973; Näätänen et al., 1992). However, to date, this effect has been mostly studied under conditions that do not fully capture the real-life challenge of attention to speech. For example, the speech materials used in many studies are often comprised either of short, context-less utterances (Brungart, 2001; Humes et al., 2017) or recordings of audiobooks that are highly edited and professionally rehearsed and recorded (Fiedler et al., 2019; Fu et al., 2019), functioning more as “spoken texts” than as natural speech. In contrast, natural speech is continuous, contextual, and produced on the fly resulting in added disfluencies, pauses, and repetitions (Agmon et al., 2023).

Another non-ecological aspect of many studies is that speech is presented only auditorily, often in a dichotic manner where the audio from different talkers is presented to different ears (Cherry, 1953; Bentin et al., 1995; Aydelott et al., 2015; Brodbeck et al., 2020; Kaufman and Zion Golumbic, 2023; Makov et al., 2023). However, in many real-life situations, listeners also look at the talker that they are paying attention to; hence, target speech is often audiovisual by nature and is emitted from a central location relative to the listener. In contrast, other non-target talkers are—by default—heard but not seen (unless listeners overtly move their head/eyes), and their audio emanates from peripheral spatial locations. Accordingly, under spatially realistic audiovisual conditions, there are stark qualitative differences between the sensory features of target and non-target speech, which likely assist listeners in focusing their attention appropriately (Fleming et al., 2021). Supporting this, it has been shown that having corresponding visual input of a talker facilitates speech processing as well as selective attention (Sumby and Pollack, 1954; Grant and Seitz, 2000; Schwartz et al., 2004; Ahmed et al., 2023a,b; Haider et al., 2024; Karthik et al., 2024; Wikman et al., 2024) and also improves the precision of neural speech tracking (Zion Golumbic et al., 2013a; Crosse et al., 2015; Fu et al., 2019).

The current study is part of ongoing efforts to increase the ecological validity of selective attention research and to advance our understanding of how the brain processes and prioritizes competing speech in the type of circumstances encountered in real life (Freyman et al., 2001; Ross et al., 2007; Tye-Mmurray et al., 2016; Shavit-Cohen and Zion Golumbic, 2019; Keidser et al., 2020; Uhrig et al., 2022; Brown et al., 2023). We capitalize on the potential of the speech-tracking approach for gaining insight into how the brain encodes and represents concurrent, continuous, and natural speech stimuli (Ding et al., 2012; Mesgarani and Chang, 2012; Zion Golumbic et al., 2013b; Kaufman and Zion Golumbic, 2023). To our knowledge, only a handful of previous studies have measured neural speech tracking in a spatially real audiovisual selective attention paradigm (O’Sullivan et al., 2019; Wang et al., 2023). In one such study, O’Sullivan et al. (2019) found that it is possible to determine from the neural signal whether a listener is paying attention to the talker they are looking at or if they are “eavesdropping” and paying attention to a peripheral talker whom they cannot see. These results nicely demonstrate the dissimilarity of the neural representation of audiovisual target speech and concurrent audio-only non-target speech. However, they leave open the question of the degree to which the brain suppresses irrelevant speech and exhibits “neural bias” for preferential encoding of target speech under spatially realistic audiovisual conditions.

There is a long-standing theoretical debate about how selective attention affects the neural representation of non-target stimuli. One possibility is that non-target speech is attenuated at an early sensory level structuring (Broadbent, 1958; Treisman, 1960; Carlyon, 2004; Ding et al., 2018), and the degree to which non-target speech is represented/attenuated is thought to reflect the efficacy of selective attention. Alternatively, target and non-target speech can be co-represented at the sensory level, with selection occurring only at later stages (e.g., the level of linguistic/semantic processing; late-selection; Deutsch and Deutsch, 1963; Murphy et al., 2013). As noted, numerous studies have demonstrated reliable “neural bias” in the sensory representation of concurrent speech in dichotic listening paradigms, showing that the acoustic envelope of target speech is tracked more precisely than non-target speech (Kerlin et al., 2010; Zion Golumbic et al., 2013b; O’Sullivan et al., 2015; Fuglsang et al., 2017; Fiedler et al., 2019; Har-Shai Yahav et al., 2024). However, although this effect is robust when averaging across multiple participants, recently, Kaufman and Zion Golumbic (2023) showed that this effect was driven by ∼30% of participants, whereas the majority of participants did not show reliable neural bias but exhibited comparable neural representation for both target and non-target speech in bilateral auditory cortex. This raised the possibility that suppression of non-target speech, at least at the sensory level, may not be a necessary component of selective attention and that differences between individuals may reflect different listening strategies or capability for multiplexed listening. However, that study, like most previous work in the field, used an auditory-only dichotic listening design, which does not capture the spatial realism of real-life audiovisual contexts.

Here we sought to replicate and extend our previous work using a more ecologically valid spatially realistic audiovisual design and to study the relative representation of target and non-target speech in the brain under these circumstances. We simulated a common real-life situation in which individuals pay attention to a talker whom they can see (in this case, watching a video recording of a lecture) but also hear another talker off to the side, whom they cannot see and are asked to ignore. We presented the audio of both target and non-target in a free-field fashion, from their respective spatial locations, rather than through earphones, to ensure realistic spatial propagation of the sound. Importantly, we used unedited recordings of actual lectures delivered by academics for the general public, to preserve the natural properties of the speech. In addition, mid-way along the experiment we switched between the target and non-target talkers, to study how this affected listeners and to test the generalizability of results across talkers and over time.

We recorded participants neural activity using electroencephalography (EEG) and analyzed their neural tracking of target and non-target speech, focusing both on group averages, as is common in the field, as well as on individual-level data (Ding et al., 2012; Ding and Simon, 2012; Fuglsang et al., 2017; Rosenkranz et al., 2021; Kaufman and Zion Golumbic, 2023). We use data-driven statistics to determine the degree to which each talker is represented in the neural signal as well as the “neural bias” toward target speech. We also tested the reliability of results between the first and second half of the experiment, and tested if switching the identity of the target talker affected the pattern of neural tracking.

## Materials and Methods

### Participants

We collected data from 24 adult volunteers (16 female, 8 male), ranging in age between 19 and 34 (*M* = 23.83, SD = ±3.42). All participants were fluent Hebrew speakers with self-reported normal hearing and no history of psychiatric or neurological disorders. The study was approved by the IRB ethics board of Bar-Ilan University, and participants gave their written informed consent prior to the experiment. Participants were either paid or received course credit for participating in the experiment. Data from one participant was excluded from all analysis due to technical issues during EEG recording, therefore all further analyses are reported for *N* = 23.

### Speech stimuli

The stimuli consisted of two 20 min video recordings of a public lecture on popular science topics, one delivered by a male talker and the other by a female talker. Each video recording included the lecturer as well as the slides accompanying the talk. Both talkers gave their approval to use these materials for research purposes. Lecture videos were segmented and edited using the software Filmora (filmora.wondershare.net) and FFMPEG (www.ffmpeg.org). Lectures were cut into 63 segments ranging between 22 and 40 s each (lecture 1: *M* = 31.83, SD = ±4.2; lecture 2: *M* = 30.68, SD = 2.24). The varied lengths were necessary to ensure that the segments did not cut off the lecture mid-sentence or mid-thought. We equated the loudness of lecture segments using peak normalization, based on the average min and max dB across all segments (separately for each talker). Then, to equate the perceived loudness of the two talkers (male and female), we performed manual perceptual calibration, based on the feedback of five naive listeners. Loudness adjustment was performed using the software FFMPEG and Audacity (version 3.2.1; www.audacityteam.org). Ultimately, the experiment included 42 segments from the start and end of each lecture (21 each), and we discarded the content from the middle of the lecture (see experimental procedure).

### Experimental procedure

**Figure 1.**
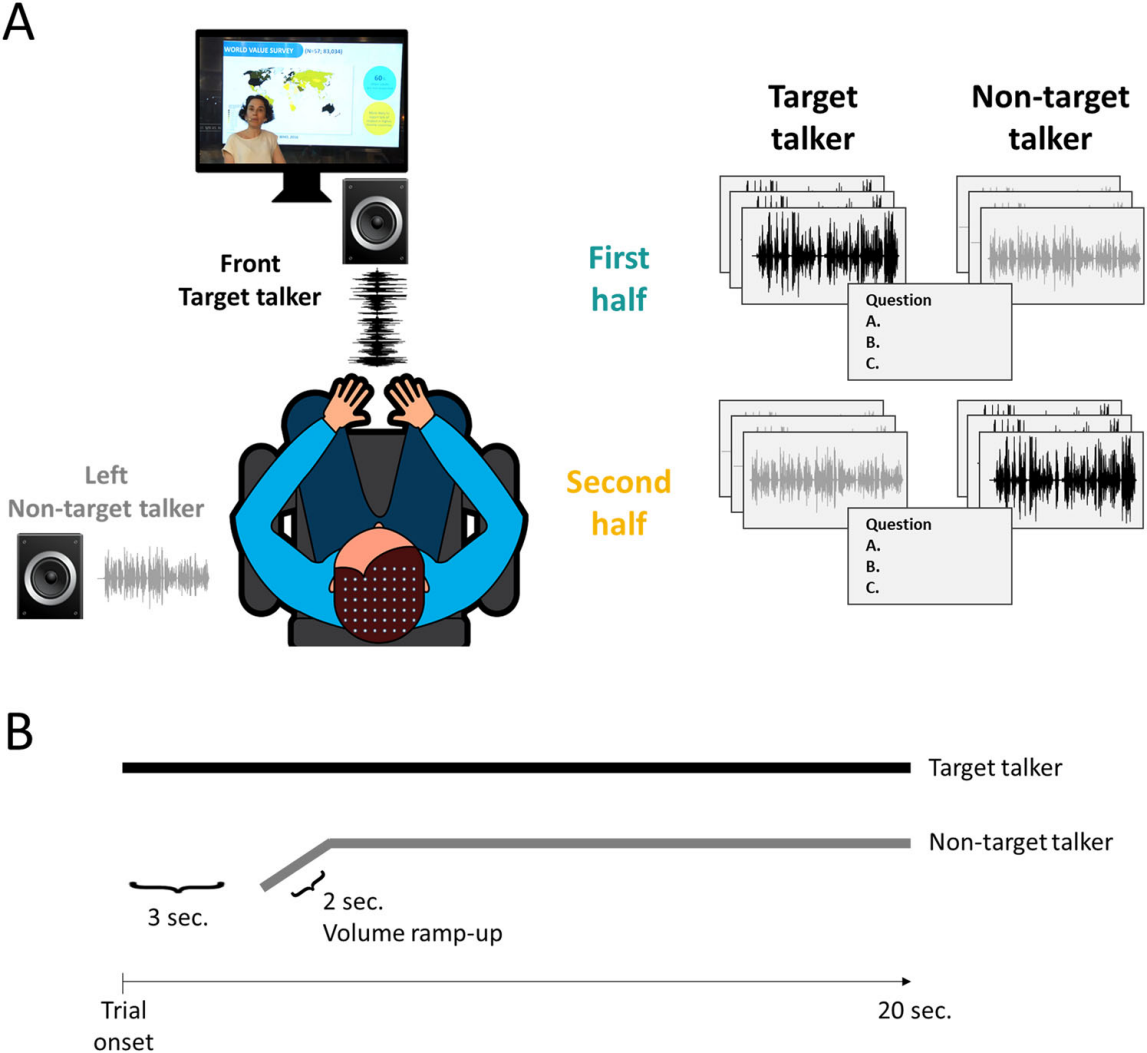
Experimental setup. ***A***, Two lectures were presented simultaneously, with one lecture (target talker) displayed on the screen and audio emitted through the front loudspeaker. The other lecture (non-target talker) was played audio-only through the left loudspeaker. Participants were instructed to focus their attention on the lecture presented on the screen. Critically, in the middle of the experiment, the stimuli switched such that the lecture that was played from the side and had been non-target in the first half, was presented as a video in the second half, and became the target talker, whereas the target talker from the first half was presented in the second half from a loudspeaker on the side and became non-target. Participants answered comprehension questions regarding the target lecture after every three trials. ***B***, Single trial illustration. Target speech began at trial onset, and non-target speech began 3 s after onset and included a 2 s volume ramp-up.

The experiment was programmed and presented to participants using the software OpenSesame (Mathôt et al., 2012). Participants were seated on a comfortable chair in a sound-attenuated booth and were instructed to keep as still as possible. Participants viewed a video lecture (target), presented segment-by-segment, on a computer monitor in front of them with the lecture audio presented through a loudspeaker placed behind the monitor. They were instructed to pay attention to the video and after every three segments, were asked three multiple-choice comprehension questions, regarding the content of the recent segments (one question per segment, four possible answers). Participants received feedback regarding the correctness of their responses and indicated via button press when they were ready to continue to the next segment. In addition to the target lecture, audio from an additional non-target lecture was presented through a loudspeaker placed on their left side (Fig. 1*A*). Segments of non-target speech began 3 s after the onset of the target speech and included a volume ramp-up of 2 s (Fig. 1*B*). Both loudspeakers were placed at the same distance from participants’ head (∼95 cm). We chose to present non-target speech only from the left side, rather than counterbalanced across both sides, to ensure sufficient amount of data for TRF estimation, without doubling the experiment length.

In the middle of the experiment, the stimuli were switched: The lecture that had been the non-target became the target lecture and was presented as a video in the second half, whereas the lecture that had been the target was presented as audio-only from a loudspeaker on the left and was the non-target (Fig. 1*A*). Importantly, different portions of each lecture were presented in each half of the experiment. When a lecture was designated as the target, it started from the beginning to ensure optimal comprehension and continued for 21 consecutive segments. When a lecture was designated as the non-target (and presented only auditorily), the last 21 segments of the lecture were played, also in consecutive order. In this way, segments from each lecture served both as target and non-target speech in different parts of the experiment (thus sharing the talker-specific attributes and general topic), but none of the content was repeated. The order of the starting lecture (male/female talker) was counterbalanced across participants, and participants were not informed in advance about the switch. Audio from on-ear microphones and eye movements were also recorded during the experiment, but their analysis is outside the scope of this study.

### EEG data acquisition

EEG was recorded using a 64 Active Two system (BioSemi; sampling rate, 2,048 Hz) with Ag-AgCl electrodes, placed according to the 10–20 system. Two external electrodes were placed on the mastoids and served as reference channels. Electrooculographic (EOG) signals were simultaneously measured by four additional electrodes, located above and beneath the right eye and on the external side of both eyes.

### Behavioral data analysis

Behavioral data consisted of accuracy on the comprehension questions asked about each segment. These values were averaged across trials, separately for each half of the experiment, and for each participant. We used a two-tailed paired *t* test to evaluate whether accuracy rates differed significantly before and after the talker switch (first and second half).

### EEG preprocessing and speech tracking

EEG preprocessing and analysis were performed using the Matlab-based FieldTrip toolbox (Oostenveld et al., 2011), as well as custom-written scripts. Raw data was first rereferenced to the linked left and right mastoids and was bandpass filtered between 0.5 and 40 Hz (fourth-order zero-phase Butterworth filter). Data were then visually inspected and gross artifacts (that were not eye movements) were removed. Independent component analysis (ICA) was performed to identify and remove components associated with horizontal or vertical eye movements as well as heartbeats (identified through visual inspection). Any remaining noisy electrodes that exhibited either extreme high-frequency activity or low-frequency drifts were replaced with the average of their neighbors using an interpolation procedure (ft_channelrepair function in the FieldTrip toolbox). The clean data was then cut into trials, corresponding to portions of the experiment in which a single segment of the lecture was presented. These were divided according to which half of the experiment they were from—before and after switching the target talker (first and second half).

To estimate neural responses to the speech from the two simultaneous lectures, we performed speech-tracking analysis, using both an encoding and a decoding approach. We estimated multivariate linear Temporal Response Functions (TRFs) using the mTRF MATLAB toolbox (Crosse et al., 2016), which constitutes a linear transfer function describing the relationship between a particular feature of the stimuli (S) and the neural response (R) recorded when hearing it.

Here S was a matrix composed of the broadband envelopes of the two speech stimuli presented in each trial, and they were treated as separate regressors in a single multivariate regression model. Envelopes were extracted using an equally spaced filterbank between 100 and 10,000 Hz, with 20 frequency bands, based on Liberman's cochlear frequency map (Liberman, 1982). The narrowband filtered signals were summed across bands after taking the absolute value of the Hilbert transform for each one, resulting in a broadband envelope signal. The R used here was the continuous cleaned EEG data, bandpass filtered again between 1 and 20 Hz (fourth-order zero-phase Butterworth filter), since the speech-tracking response consists mostly of low-frequency modulations. S and R were aligned in time and downsampled to 100 Hz for computational efficiency. The five first seconds of each trial were excluded from data analysis, due to the differences in onset and ramp-up period (Fig. 1*B*). In addition, the first trial and the trial immediately after the talker switch were omitted from data analysis, to avoid confounding effects associated with attentional ambiguity and adjustment to the new target talker. This resulted in a total of 40 trials analyzed (20 per half), each ∼30 s long (see above).

Univariate and multivariate encoding and decoding models were optimized separately for each half of the experiment. In the encoding approach, linear TRFs are estimated reflecting the neural response at each electrode for each of the two simultaneously presented stimuli, and the predictive power of the model reflects how it predicts the actual neural response recorded. In the decoding approach, the neural data is used to reconstruct the envelope of each speech stimulus. TRF predictive power values (encoding) and reconstruction accuracies (decoding) were assessed using a leave-one-out cross-validation protocol. In each iteration, all trials except one were randomly selected to train the model (train set), which was then used to predict either the neural response at each electrode (encoding) or the two speech envelopes (decoding) in the left-out trial (test set). The predictive power of the encoding model is the Pearson's correlation (*r* value) between the actual neural response in the left-out trial and the response predicted by the model. The decoding reconstruction accuracies is calculated separately for the two speech stimuli presented in the left-out trial (target and non-target speech) and is the Pearson's correlation (*r* value) between the reconstructed envelope of each and the actual envelope. The reported TRFs, predictive power values, and reconstruction accuracies are the averages across all iterations.

Encoding TRFs were calculated over time lags ranging from −150 (pre-stimulus) to 450 ms, and the decoding analysis used time lags of 0–400 ms, as is customary in similar analyses (Crosse et al., 2016). To prevent overfitting of the model, a ridge parameter was chosen as part of the cross-validation process (*λ* predictive power). This parameter significantly influences the shape and amplitude of the TRF and therefore, rather than choosing a different *λ* for each participant (which would limit group-level analyses), a common *λ* value was selected for all participants, which yielded the highest average predictive power, across all channels and participants (see also Har-shai Yahav et al., 2024; Kaufman and Zion Golumbic, 2023). For both the encoding and decoding models, this optimal value was *λ* = 1,000 a. Note that decoding results were highly similar for *λ*'s that were optimized separately for each participant (data not shown).

### EEG statistical analysis

#### Group-level statistics

The statistical significance of the predictive power and reconstruction accuracy of the encoding and decoding models were evaluated using permutation tests. For this, we repeated the encoding/decoding analysis procedure on shuffled S-R data where speech envelopes presented in one trial (S) were paired with the neural response recorded in a different trial (mismatched R). This procedure was repeated 100 times, yielding a null distribution of predictive power/reconstruction accuracy values that could be obtained by chance. The real data was then compared with this null distribution and if it fell, within the top 5 percentile was considered statistically significant. To compare the speech-tracking response across conditions, we conducted a 2 × 2 ANOVA a Bayesian factor analysis with repeated measures to compare the predictive power and reconstruction accuracies obtained for target versus non-target speech, in each half of the experiment (before vs after the target talker switch; JASP-Team, 2022; version 0.16.3; prior distribution parameters: Uniform Cauchy distribution with a scale parameter of *r* = 0.5, random effects *r* = 1, scale covariates *r* = 0.354).

To test the generalizability of the speech-tracking patterns between the two halves of the experiment, we also tested how well decoders that were trained on data from one half of the experiment (either on target or non-target speech) could be used to accurately predict the stimuli presented in the other half of the experiment based on the neural data.

#### Individual-level statistics

**Figure 2.**
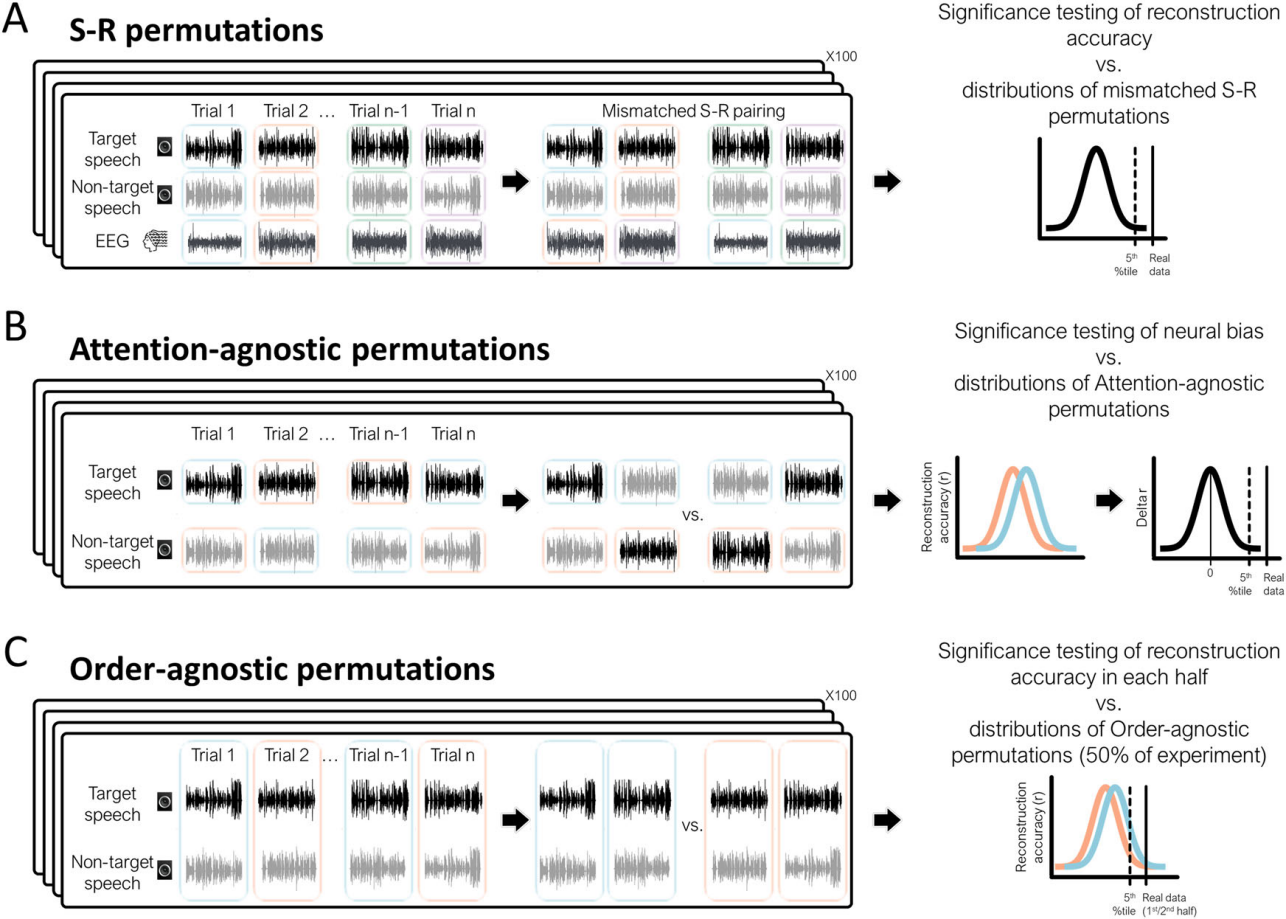
Data-driven permutation tests for individual-level statistics. Three permutation tests were designed to assess statistical significance of different results in individual participants. The black rectangles in all panels show the original data organization on the left and the relabeling for permutations test on the right. ***A***, S-R permutation test. In each permutation, the pairing between acoustic envelopes (S) and neural data responses (R) was shuffled across trials such that speech envelopes presented in one trial (both target and non-target speech) were paired with the neural response (R) from in a different trial. This yields a null distribution of reconstruction accuracies that could be obtained by chance, to which the real data can be compared (right). ***B***, Attention-agnostic permutation test. In each permutation, the target and non-target speech stimuli were randomly relabeled to create attention-agnostic regressors that contain 50% target speech and 50% non-target speech. The reconstruction accuracy for each regressor was estimated and the difference between them is used to create a null distribution to which the neural-bias index can be compared (right). ***C***, Order-agnostic permutation test. In each permutation, trials were randomly relabeled and separated into two order-agnostic groups consisting of 50% trials from the first half of the experiment and 50% trials from the second half. The reconstruction accuracy for each group of trials was estimated, and the difference between them is used to create a null distribution to which the real data from each half of the experiment can be compared (right).

Statistical analysis of individual-level data focused only on reconstruction accuracies (decoding approach), since this approach integrates responses across all electrodes, yielding a simpler metric for statistical analysis and avoiding multiple comparisons. We conducted a series of permutation tests to obtain data-driven statistics in each individual participant, designed to address different questions, as illustrated in Figure 2.

First, we assessed whether the speech reconstruction accuracies obtained for both target and non-target speech were significantly better than those that could be obtained chance. To this end, we used S-R permutations, similar to those used for group-level statistics, in which we shuffled the pairing between acoustic envelopes (S) and neural data responses (R; Fig. 2*A*). Reconstruction values were assessed in 100 permutations of mismatched S-R combinations, yielding a null distribution from which we derived a personalized chance-level value for target and non-target speech, for each participant (the top 5 percentile of the null distribution).

Second, we assessed whether the difference in the reconstruction accuracy for the target and non-target talker could reliably be attributed to their task relevance (referred to as the “Neural-Bias index”). For this, we followed the procedure introduced by Kaufman and Zion Golumbic (2023) to create an “attention-agnostic” null distribution of neural-bias indices (Fig. 2*B*). Specifically, for each participant we created 100 permutations in which the two speech stimuli were randomly relabeled so that the stimuli represented in each regressor was 50% target and 50% non-target. Multivariate decoders were trained on this relabeled data and reconstruction accuracy values were estimated for each regressor, and difference between them was used to create an attention-agnostic distribution of differences between reconstruction accuracies. The real neural-bias index for each participant was normalized (*z*-scored) relative to this null distribution, and participants with a *z*-score >1.64 were considered as exhibiting a significance neural bias toward the target speech (*p* < 0.05, one-tailed). We chose this normalization continuous approach, rather than using a cutoff value, which allows us to present the distribution of neural-bias values across participants. We note that the approach used here to assess differences in speech tracking of target and non-target here differs from the auditory attention-decoding (AAD) approach used in similar studies to identify which of two speech stimuli belongs to the target talker (Mirkovic et al., 2015; O’Sullivan et al., 2015, 2019; Fuglsang et al., 2017; Teoh and Lalor, 2019). In those studies, a decoder trained only on target speech is used to predict the envelope of non-target speech (and vice versa), which assesses the similarity/differences between the decoders estimated for each stimulus. However, this approach is less appropriate in the current study, where we were interested in assessing how accurately each speech stimulus is represented in the neural signal, even if the spatiotemporal features of their decoders are different (see Extended Data Fig. 6-1 for a direct comparison of these approaches and discussion of their utility for different purposes).

The two analyses described above (Fig. 2*A*,*B*) were performed using data from the entire experiment, as well as data from each half of the experiment separately. We then performed a third permutation test to assess whether speech reconstruction accuracies and the Neural-Bias index differed significantly in the two halves of the experiment, i.e., before versus after the talker switch. For this, we conducted an “order-agnostic” permutation test where trials were randomly relabeled so that the data included in each regressor were 50% from the first half of the experiment and 50% from the second half (Fig. 2*C*). Multivariate decoders were trained on this relabeled data and reconstruction accuracy values were estimated for each regressor, and this procedure was repeated 100 times, yielding a null distribution. A participant was considered as showing a significant difference in neural tracking of either the target or non-target talker, or different in neural bias, if their real data fell in the top 5 percentile of the relevant null distribution.

## Results

### Behavioral data

**Figure 3.**
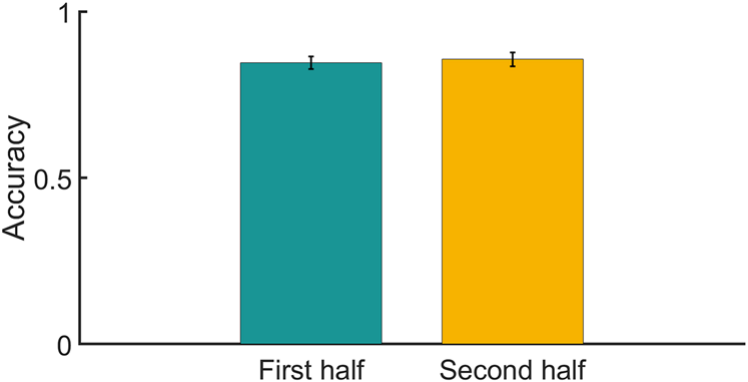
Behavioral results. Averaged accuracy rates across trials and participants, for multiple-choice comprehension questions, separately for the first (green) and second (yellow) half of the experiment. Error bars denote SEM across participants.

Accuracy in answering comprehension questions about the content of the lecture was significantly above chance (*M* = 0.845, SD = ±0.076; *t*_(22)_ = 32.178, *p* < 10^−20^). No significant differences in performance were observed between the first half (*M* = 0.846, SD = ±0.09) and second half (*M* = 0.856, SD = ±0.1) of the experiment (*t*_(22) _= −0.42, *p* = 0.68), as shown in Figure 3.

### Speech-tracking analysis

#### Group-level results

**Figure 4.**
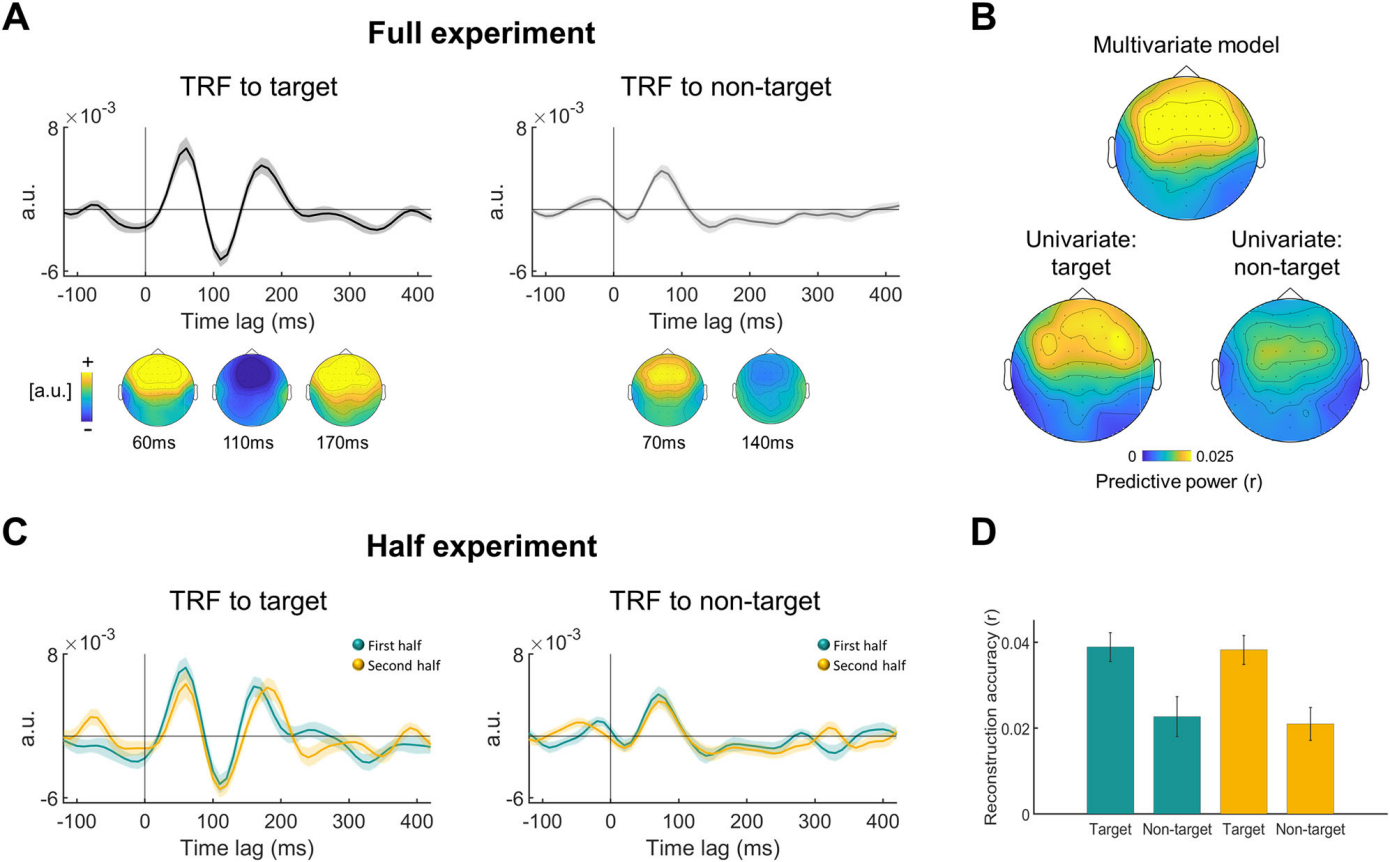
Neural bias: group-level results. ***A***, TRF encoding models across all experimental trials, plotted from electrode “Fz,” separately for target and non-target speech. Shaded highlights denote SEM across participants (top). Topographic distribution of the TRF main peaks, plotted separately for target and non-target speech (bottom). ***B***, Topographic distribution of predictive power values (Pearson's *r*) of the encoding model, averaged across participants, separately for multivariate (top) and univariate (bottom) analysis. ***C***, TRF encoding models for the first half (green) and second half (yellow) of the experiment, plotted from electrode “Fz,” separately for target and non-target speech. Shaded highlights denote SEM across participants. ***D***, Speech reconstruction accuracies for the first and second half of the experiment, for both target and non-target speech. Error bars denote SEM across participants. Extended Data Figure 4-1 is supporting Figure 4.

Figure 4 shows the results of the encoding approach. TRF models were estimated for target and non-target speech, trained on data from the entire experiment and also separately on each half of the experiment (before and after talker switch). As expected, the predictive power followed the common centro-frontal topography characteristic of auditory responses in EEG (Fig. 4*A*) and was significant compared with a null distribution (*p* < 0.01). The TRF for target speech showed three prominent peaks—at 60, 110, and 170 ms—which is in line with previous studies and are thought to reflect a cascade of information flow from the primary auditory cortex to associative and higher-order regions (Brodbeck et al., 2018b). The TRF for non-target speech was also robust but showed only a single positive peak, ∼70 ms, which likely reflects its early sensory encoding, but not the two later peaks. Although the TRFs estimated for target and non-target speech are not directly comparable (due to the many sensory differences between them, i.e., location, audiovisual vs audio only), they did differ in the amount of variance they explained in the neural signal (predictive power of univariate models for target vs non-target, averaged across all electrodes; *F*_(22)_ = 11.5, *p* = 0.003) and including both regressors in a multivariate model explained significantly more of the variance in the neural signal than either univariate model alone (*t* test between average predictive power: multivariate vs target only: *t* = 2.97, *p* = 0.007; multivariate vs non-target only: *t* = 5.9, *p* < 10^−5^; Fig. 4*B*).

10.1523/ENEURO.0132-24.2025.f4-1 Figure 4-1 **Group-level Spectral analysis.** Comparison of the power spectrum of the EEG between the first and second half of the experiment. We calculated the spectral power density using a multitaper fast-fourier transform (FFT; as implemented in Fieldtrip), separately for the data from each half of the experiment. Shown in the figure is the power spectrum averaged over 9 centro-parietal electrodes (marked in the subplot on the top-left). A clear peak is seen in the low alpha-range (7-9  Hz) in both halves of the experiment, which was maximal at centro-parietal electrodes (shown in the topographies). However, a paired t-test revealed no significant difference between alpha power in the first vs. second half of the experiment [t(22) = 1.25, p = 0.22], which might have been expected as an index of fatigue or reduced attention over time (e.g. Yu et al., 2021). Download Figure 4-1, TIF file.

The TRFs estimated separately in the first and second half of the experiment for target and non-target speech were highly similar in their spatiotemporal properties, and no significant differences between them were found (Fig. 4*C*). An ANOVA comparing the reconstruction accuracies for target versus non-target speech across both halves of the experiment revealed a main effect of task relevance (target vs non-target: *F*_(1,22)_ = 28.3, *p* < 0.001) but no main effect of half (*F*_(1,22)_ = 0.12, *p* = 0.73) or interaction between the factors (half × talker: *F*_(1,22)_ = 0.87, *p* = 0.87). These results were confirmed using a Bayesian ANOVA which indicated that the main effect of task relevance could be embraced with high confidence and explains most of the variance (BF_inclusion_ = 317, *p* = 0.002) but there was no reliable difference between the two halves or interaction (BF_inclusion_ = 0.26 and BF_inclusion_ = 0.27, respectively, both *p*s > 0.7).

**Figure 5.**
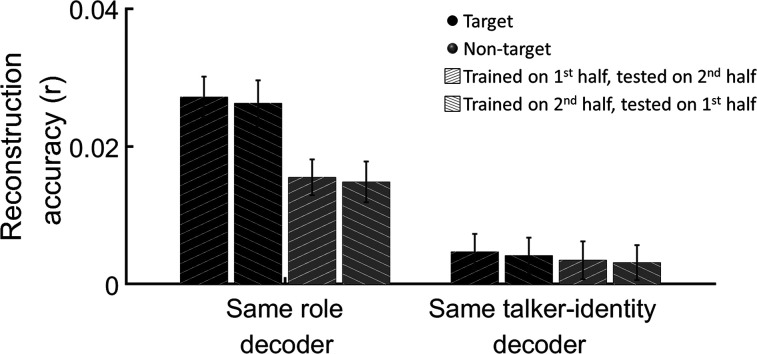
Generalizability across talkers and time. Reconstruction accuracies for decoders trained on data from one half of the experiment (either on target or non-target speech) and tested on data from the other half of the experiment, separately for same role decoders (e.g., train on target and test on target) and for same talker identity decoders (e.g., train on male talker, test on male talker).

Moreover, we found that decoders trained in one half of the experiment generalized well to the other half when tested on stimuli that shared the same task relevance (role—target/non-target) but did not generalize well to stimuli that shared the same talker identity but had different roles in the two halves of the experiment (Fig. 5). Moreover, the modulation of reconstruction accuracy by task relevance was preserved even in this cross-over analysis. This suggests that the decoders are largely invariant to talker identity and primarily capture features related to the role of the talker in the given task and/or their sensory properties (in this case, being presented audiovisually from a central location for the target speaker).

Besides the speech-tracking analysis, we also conducted another group-level analysis of the data and inspected whether the spectral makeup of the EEG signal, and particularly power in the alpha range (8–12 Hz), differed between the first and second halves of the experiment. These results are shown in Extended Data Figure 4-1, as no significance differences were found.

#### Individual-level results

Statistical analysis of speech tracking in individual participants was three tiered, assessing the significance of: (1) reconstruction accuracies for target and non-target speech, (2) the neural-bias index, and (3) differences between the two parts of the experiment.

**Figure 6.**
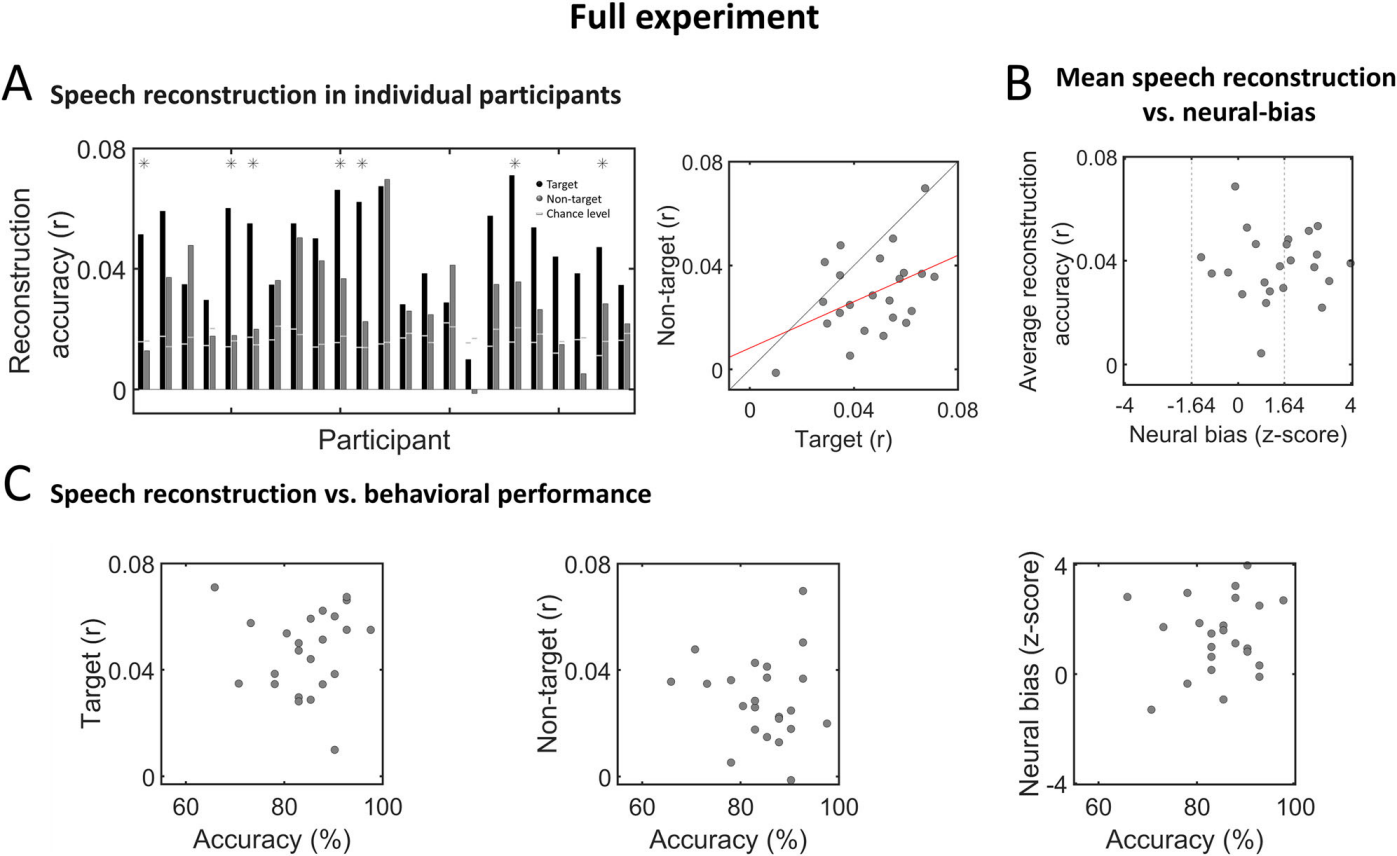
Speech reconstruction and neural bias in individual participants—full experiment. ***A***, Left, Bar graphs depicting reconstruction accuracy in individual participants for target (black) and non-target (dark gray) speech. Horizontal light gray lines represent the *p* = 0.05 chance level, derived for each participant based on data-driven S-R permutation. Asterisks indicate participants who also showed significant neural bias to target speech (see panel ***B***). Right, Scatterplot showing reconstruction accuracies for target and non-target speech across all participants. The red line represents the linear regression fit between the two variables, which was significant (Pearson's *r* = 0.43, *p* = 0.038). ***B***, Scatterplot showing the average reconstruction accuracy and neural-bias index across participants, which were not significantly correlated. Vertical dashed lines indicate the threshold for significant neural bias (*z* = 1.64, one-tailed; *p* < 0.05). ***C***, Scatterplots showing the accuracy on behavioral task versus reconstruction accuracy of target speech (left), non-target speech (middle), and the neural-bias index (right), across all participants. No significant correlations were found. Extended Data Figure 6-1 is supporting Figure 6.

Figure 6*A* (left panel) shows the reconstruction accuracies for target and non-target speech in individual participants, relative to their personal chance-level value (*p* = 0.05 cutoff, derived in a data-driven manner using S-R permutation). All but one participant showed significant reconstruction accuracy of the target speech (22/23 participants—95%), and most participants also showed higher than chance reconstruction for the non-target speech (18/23 participants—78%). Moreover, reconstruction accuracies for target and non-target speech were positively correlated, across participants (Pearson's *r* = 0.43, *p* = 0.038; Fig. 6*A*, right panel).

10.1523/ENEURO.0132-24.2025.f6-1 Figure 6-1 **Comparison of decoder-testing approaches.** Here we compare two approaches for testing the performance of decoders trained on EEG data to reconstruct the envelope of concurrently presented speech. ***Top***: The approach used and reported in the current study, in which two **Stimulus specific decoders** were trained using a multivariate approach to reconstruct the envelopes of target and non-target speech presented concurrently. The scatter-plot shows reconstruction accuracies achieved for both decoders across all participants, when tested on left-out data of the same type (i.e., how well the target decoder can reconstruct left-out target speech, and how well the non-target decoder can reconstruct left-out non-target speech). The gray line reflects the diagonal, and the red line represents the linear regression fit between the two variables which was statistically significant [data is the same as in Figure 6A]. ***Bottom***: Re-analysis of the same data using the auditory attention-decoding (AAD) approach, in which a decoder is trained only on one stimulus (e.g., on target speech), and is then tested on left-out data of the same stimulus (target) and of the other stimulus (non-target), and the two results are compared for classification purposes. The left panel shows a scatter-plot showing how well a decoder trained on target speech can reconstruct left-out target speech vs. how well it can reconstruct left-out non-target speech, across all participants. The left panel shows the same for a decoder trained on non-target speech. The gray line reflects the diagonal, and the red line represents the linear regression fit between the two variables (dashed line indicates a marginally significant regression). In this analysis, almost all dots fall either below or above the diagonal, clearly showing between reconstruction performance when a decoder is tested on data of the same type that it was trained on. This is in line with multiple studies, that propose using this approach for practical applications, such as controlling a neuro-steered hearing device (Henshaw and Ferguson, 2013; O’Sullivan et al., 2015; Kidd, 2017; Geirnaert et al., 2022; Roebben et al., 2024). Given the qualitative sensory differences between target and non-target speech in the spatially-realistic audiovisual setup used (e.g., spatial location, audio/audio-visual presentation etc.), it is not very surprising that decoders trained on these stimuli would differ from each other. However, we posit that this approach is less appropriate in the current study, where the goal was not just to distinguish between the two stimuli, but to test whether target speech is represented more robustly in the neural data than non-target speech, a pattern that is considered a signature of ‘selective attention’ - i.e., enhancement of target speech and/or suppression of non-target speech (Kerlin et al., 2010; Ding et al., 2012; Zion Golumbic et al., 2013b; O’Sullivan et al., 2015; Fiedler et al., 2019). For this purpose, we believe that is it more appropriate to optimize decoders for each stimulus separately (thus accounting for their differences in properties, e.g. differences in spatial location or speaker characteristics), and then assess how well each one performs for predicting the stimulus it was trained on (the model’s goodness-of-fit/predictive power/accuracy). Using this approach, if we find that both decoders perform very well – this indicates that both stimuli are represented with similar precision in the neural response. Conversely, finding that the decoder for one stimulus outperforms the other can be interpreted as superior or more detailed neural encoding of that stimulus relative to the other, effects that have been associated with better intelligibly and/or higher levels of attention to this stimulus (Best et al., 2008; Lin and Carlile, 2015; Getzmann et al., 2017; Teoh and Lalor, 2019; Uhrig et al., 2022; Orf et al., 2023). Download Figure 6-1, TIF file.

In Figure 6*B*, we compare the average reconstruction accuracy from each participant to their “Neural-Bias index,” which reflects the difference in reconstruction accuracy for the target vs non-target speech (normalized relative “target-agnostic” permutations of the data). When using a cutoff of *z* > 1.64 (*p* < 0.05, one-tailed), only 10/23 participants (43%) showed a significant neural-bias index, and if we use a less conservative threshold of *z* > 1 (*p* < 0.15, one-tailed), this proportion changes only slightly to 13/23 participants (56%). Interestingly, the reconstruction accuracies themselves and the neural-bias index were not correlated with each other (Pearson's *r* = −0.017, *p* = 0.94), suggesting that these metrics are independent.

We further tested whether performance on the behavioral tasks (answering comprehension questions) was correlated with reconstruction accuracy of either speech stimuli or the neural-bias index; however, none of the brain–behavior correlations were significance (target reconstruction accuracy vs behavior: *r* = 0.032, *p* = 0.88; non-target reconstruction accuracy vs behavior: *r* = −0.088, *p* = 0.69; neural bias vs behavior: *r* = 0.14, *p* = 0.5; Fig. 6*C*).

**Figure 7.**
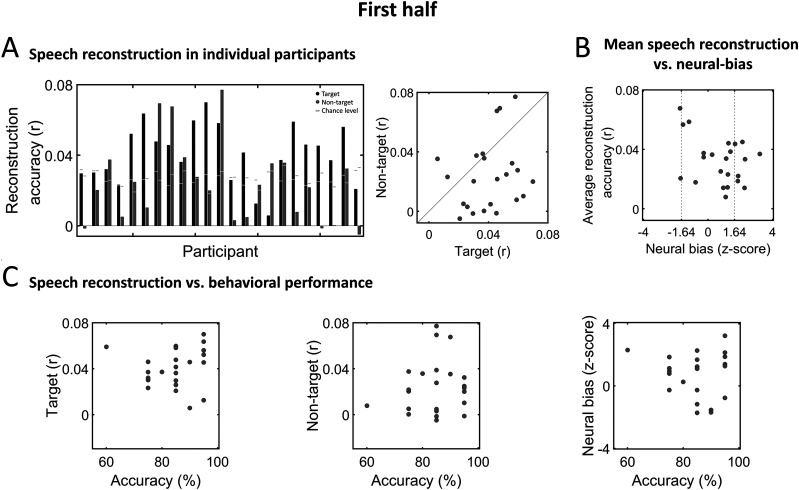
Speech reconstruction and neural bias in individual participants—first half of experiment. ***A***, Left, Bar graphs depicting reconstruction accuracy in individual participants for target (black) and non-target (dark gray) speech. Horizontal light gray lines represent the *p* = 0.05 chance level, derived for each participant based on data-driven S-R permutation. Right, Scatterplot showing reconstruction accuracies for target and non-target speech across all participants. ***B***, Scatterplot showing the average reconstruction accuracy and neural-bias index across participants, which were not significantly correlated. Vertical dashed lines indicate the threshold for significant neural bias (*z* = 1.64, one-tailed; *p* < 0.05). ***C***, Scatterplots showing the accuracy on behavioral task versus reconstruction accuracy of target speech (left), non-target speech (middle), and the neural-bias index (right), across all participants. No significant correlations were found.

**Figure 8.**
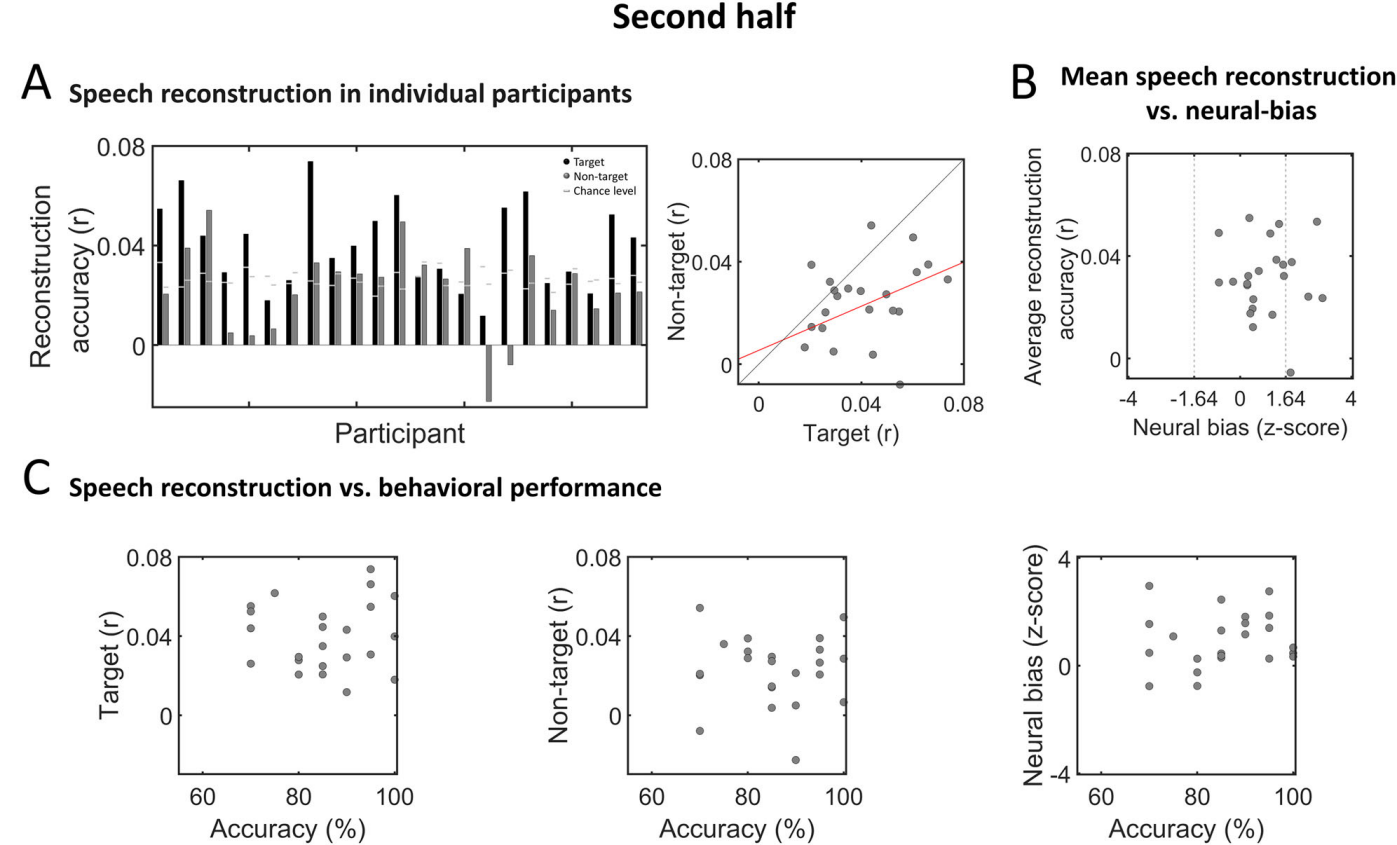
Speech reconstruction and neural bias in individual participants—second half of experiment. ***A***, Left, Bar graphs depicting reconstruction accuracy in individual participants for target (black) and non-target (dark gray) speech. Horizontal light gray lines represent the *p* = 0.05 chance level, derived for each participant based on data-driven S-R permutation. Right, Scatterplot showing reconstruction accuracies for target and non-target speech across all participants. The red line represents the linear regression fit between the two variables, which was significant (Pearson's *r* = 0.4, *p* = 0.048). ***B***, Scatterplot showing the average reconstruction accuracy and neural-bias index across participants, which were not significantly correlated. Vertical dashed lines indicate the threshold for significant neural bias (*z* = 1.64, one-tailed; *p* < 0.05). ***C***, Scatterplots showing the accuracy on behavioral task versus reconstruction accuracy of target speech (left), non-target speech (middle), and the neural-bias index (right), across all participants. No significant correlations were found.

Figures 7 and 8 depict the results of the same analyses shown in Figure 6 but conducted separately on data from each half of the experiment. Here, speech reconstruction was significant in a fewer proportion of participants, with 17/23 (74%) showing significant reconstruction of target speech in both the first and second half (although these were not necessarily the same participants) and 10/23 (52%) or 12/23 (43%) participants showing significant reconstruction of non-target speech in the first and second half of the experiment, respectively (Figs. 7*A*, 8*A*). Reconstruction accuracy of target and non-target speech were not significantly correlated in the first half of the experiment (Pearson's *r* = 0.2, *p* = 0.33) but were in the second half (Pearson's *r* = 0.4, *p* = 0.048).

When evaluating the neural-bias index of individual participants, we found that only 7/23 (30%) and 5/23 (21%) showed significantly better reconstruction accuracy for the target versus non-target speech (*z* > 1.64, *p* < 0.05, one-tailed; first and second half, respectively). As observed for the full experiment, here too reconstruction accuracy was not correlated with the neural-bias index in either half (first half: *r* = −0.37, *p* = 0.08; second half: *r* = −0.026, *p* = 0.9; Figs. 7*B*, 8*B*), nor were any brain–behavior correlations significant (first half: target reconstruction accuracy vs behavior: *r* = 0.095, *p* = 0.67; non-target reconstruction accuracy vs behavior: *r* = 0.14, *p* = 0.54; neural bias vs behavior: *r* = −0.075, *p* = 0.73; Fig. 7*C*; second half: target reconstruction accuracy vs behavior: *r* = 0.05, *p* = 0.83; non-target reconstruction accuracy vs behavior: *r* = −0.007, *p* = 0.97; neural bias vs behavior: *r* = 0.1, *p* = 0.64; Fig. 8*C*).

**Figure 9.**
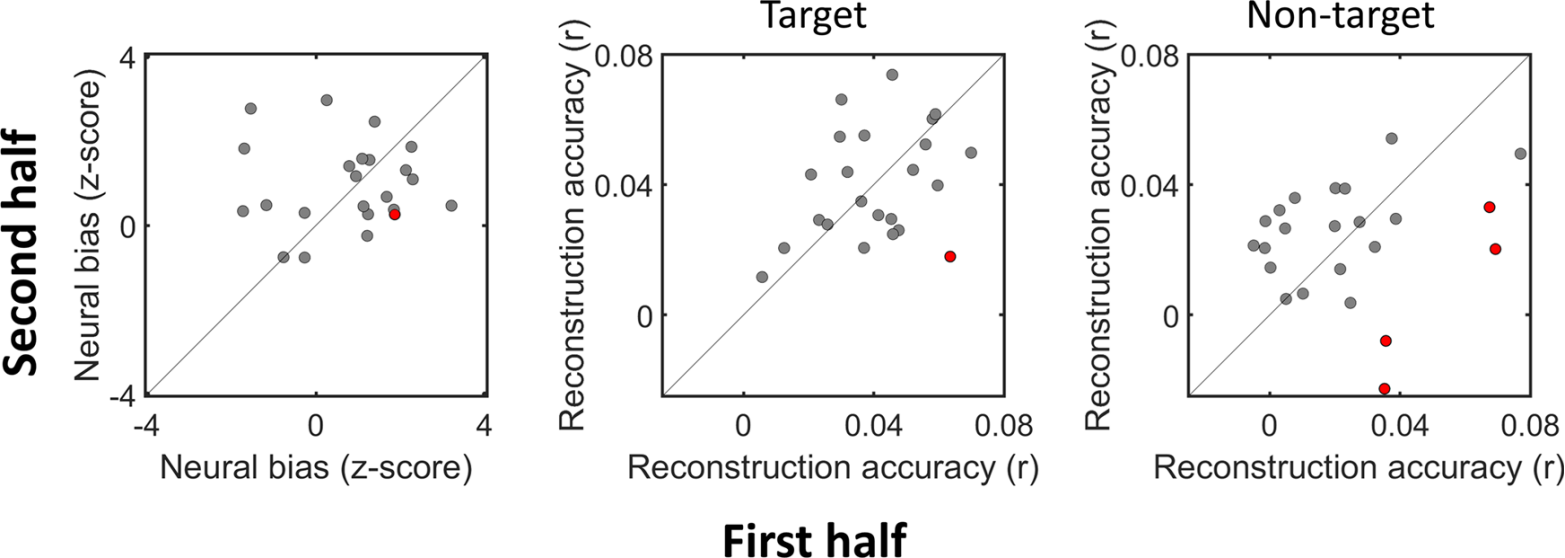
First versus second half of experiment. Scatterplots showing the neural-bias index (left), target speech (middle), and non-target speech reconstruction accuracies (right) across all participants, in the first versus second half of the experiment. No significant correlations were found for any of the measures. Participants for whom significant difference were found between the two halves (based on an order-agnostic permutation test) are marked in red.

Last, we compared the speech reconstruction accuracies and neural-bias indices obtained in each half of the experiment but found that none of these measures were significantly correlated between the first and second half of the experiment (neural bias: Pearson's *r* = −0.005, *p* = 0.98, target speech: Pearson's *r* = 0.34, *p* = 0.11, non-target speech: Pearson's *r* = 0.17, *p* = 0.44; Fig. 9). Only a handful of participants showed above-chance differences between the first and second half of the experiment in these metrics, relative to a distribution of order-agnostic permuted data (Fig. 9, red); however, these may represent false positives due to multiple comparisons.

## Discussion

Here we studied the neural representation of target and non-target speech in a spatially realistic audiovisual setup, at both the group-level and in individual participants. The group-level results are in line with results from previous two-talker experiments that used less ecological paradigms (e.g., dichotic listening, scripted speech materials etc.), namely, that the acoustic envelope of target speech is represented more robustly than that of non-target speech (Kerlin et al., 2010; Ding et al., 2012; Mesgarani and Chang, 2012; Zion Golumbic et al., 2013b; O’Sullivan et al., 2015; Fuglsang et al., 2017; Brodbeck et al., 2018b; Fiedler et al., 2019; Niesen et al., 2019; Har-shai Yahav and Zion Golumbic, 2021; Kaufman and Zion Golumbic, 2023; Straetmans et al., 2024). We also show that neural tracking is invariant to talker identity and does not change significantly over the course of the experiment (in magnitude or spatiotemporal features of the decoder). This supports the robustness of this measure for use in free-field audiovisual contexts, laying the foundation to extend scientific investigation of selective attention to more realistic environments (Parsons, 2015; Risko et al., 2016; Brown et al., 2023; Levy et al., 2024).

However, examination of individual-level results revealed that the neural bias observed at the group level is not seen consistently in all, or even in most, listeners. Fewer than half of the participants showed significant neural bias for target speech, whereas in most participants non-target speech could be reconstructed just as well as target speech from the neural signal. Importantly, reconstruction accuracy and the neural-bias index were not correlated, indicating that variability across participants cannot be trivially explained by poor speech tracking or low SNR. Moreover, neither the reconstruction accuracy of target or non-target speech nor the neural-bias index were significantly correlated with performance, suggesting they carry limited behavioral consequences. These results are similar to those reported in a previous MEG study (Kaufman and Zion Golumbic, 2023), which taken together lead us to suggest that although speech-tracking metrics are useful for studying selective attention at the group level, they may fall short as “neural-markers of selective attention” at the individual level. Below we discuss the potential implications of these results for understanding the different strategies listeners may employ to deal with concurrent speech in realistic multitalker contexts.

### Group-level effects: highly robust and generalizable

The group-level TRFs, derived separately for target and non-target speech using an encoding approach, represent spatiotemporal filters that capture the neural responses to each of the two concurrent talkers (Ding and Simon, 2012; Power et al., 2012; Zion Golumbic et al., 2013b; Fuglsang et al., 2017; Fiedler et al., 2019; Kaufman and Zion Golumbic, 2023). They share similar frontocentral topographical distribution, which is typical of auditory responses; however, they differ in their time course. The TRF for target speech contains three prominent peaks—roughly at 60, 110, and 170 ms—which is in line with previous studies and are thought to reflect a cascade of information flow from primary auditory cortex to associative and higher-order regions (Brodbeck et al., 2018b; Chen et al., 2023). Conversely, the TRF for non-target speech showed only a single positive peak, ∼70 ms, which likely reflects its early sensory encoding, but not the two later peaks. Past studies have reported mixed results regarding the temporal similarity of TRFs for target and non-target speech, some showing similar time courses albeit with reduced amplitudes for non-target speech (Kerlin et al., 2010; Ding et al., 2012; Zion Golumbic et al., 2013b; O’Sullivan et al., 2015; Fiedler et al., 2019; Kaufman and Zion Golumbic, 2023) and others showing that the later TRF peaks are not present for non-target speech (Jaeger et al., 2020; Har-Shai Yahav et al., 2024). It is likely that differences in spatiotemporal characteristics of TRFs for target and non-target speech are affected both by the specific perceptual attributes of the stimuli themselves (e.g., audiovisual vs audio presentation, their different spatial location and consequent reverberation pattern) as well as by their task-related role (target vs non-target). In the spatially realistic experimental design used here, these factors are inherently confounded, just as they are under real-life conditions in which listeners look at the talker that they are trying to pay attention to. A previous study by O’sullivan et al. (2019) and reanalyzed by Ahmed et al. (2023a) attempted to dissect the specific contribution of selective attention versus audiovisual input by including a condition in which participants watched video of a talker that they had to ignore and attended to a talker who they could not see. However, it is not clear to what degree such a highly artificial design (which is also extremely cognitively demanding) is representative of the mechanisms that listeners use when processing speech under natural circumstances. Instead, here, rather than trying to assert whether the differences in TRF are due to “selective attention per se” or to “perceptual differences,” we accept that in real life these often go together. We posit that as selective attention research progresses to more ecologically valid contexts, these factors cannot (and perhaps need not!) be teased apart but rather should be considered as making inherently joint contributions to behavior and neural responses. Arguably, in the aspiration to empirically dissociate between “pure” effects of perception and attention in ecological contexts may be inadequate, and we believe that one of the great challenges facing our field today is to reconsider and redefine the relationship between these constructs, if we strive to truly understand how the brain operates under real-life conditions (Anderson, 2011; Risko et al., 2016; Schotter et al., 2025).

Regarding the comparison between the first and second half of the experiment, we found similar TRFs and reconstruction accuracies, both for target and for non-target speech. This indicates that listeners were highly effective at adapting their neural encoding after the mid-way shift in the identity of the target talker and the topic of the lecture. It also demonstrates the robustness of EEG-based speech-tracking measures and of the neural bias toward target speech when using roughly 10 min of data (at least at the group level). When designing this study, we postulated that neural tracking and/or neural bias to the target speech might be worse in the second half of the experiment, either due to fatigue (Moore et al., 2017; Jaeger et al., 2020) or due to higher cognitive interference of the non-target talker who had previously been the target talker (Johnsrude et al., 2013; Har-Shai Yahav et al., 2024). The finding that here the switch did not carry a behavioral or neural processing cost is testimony to the high adaptability and flexibility of the attentional system, which does not “get stuck” on processing previously relevant features but updates its preferences according to changing task demands (Kiesel et al., 2010; Koch and Lawo, 2014; Agmon et al., 2021; Kaufman and Zion Golumbic, 2023). This result is somewhat in contrast to some previous findings showing that speech processing was adversely affected by attention switching. For example, a recent study similar to ours (albeit using auditory-only stimuli) found that a previously attended stream posed more of an interference to behavior task compared with a consistently task-irrelevant stream (Orf et al., 2023). Other studies have also demonstrated reduced speech processing, decreased intelligibility, and impaired recall of specific details, resulting from attention switching between talkers (Best et al., 2008; Lin and Carlile, 2015; Getzmann et al., 2017; Teoh and Lalor, 2019; Uhrig et al., 2022). However, in those studies, the switches occurred on a per-trial basis, which likely creates more opportunities for confusion relative to the current study where the target talker was switched only once. Admittedly, there is much yet to explore regarding attention switching between talkers, particularly under ecological conditions where switches are contextual and often initiated by the listener themselves. The current findings contribute to these efforts by demonstrating that the neural representation of target and non-target speech is invariant to talker identity and stabilizes nicely after a switch in a realistic audiovisual context.

### From group averages to individual-level responses

The vast majority of cognitive neuroscience research, and particularly when using EEG, relies on averaging results from large samples of participants to obtain group-level results. This has traditionally been motivated by the noisy nature of many EEG metrics, the variability across individuals, and the need to generalize results beyond a specific sample (Luck et al., 2000; Makeig et al., 2004; Luck, 2014). However, increasingly, there is also a desire to derive reliable EEG-based measures from the brains of individuals—to be used, for example, to explain variability in behavioral performance and cognitive capabilities, as biomarkers for clinical symptoms, and to monitor the effectiveness of personalized interventions (O’Sullivan et al., 2017; Bednar and Lalor, 2020; Hadley and Culling, 2022; Geirnaert et al., 2024). Deriving reliable individual-level EEG-based metrics for speech processing and/or for attention has been particularly appealing, given their potential utility for clinical, educational, and technological interventions. The development of speech-tracking methods over the past decade has given hope that this metric will prove to be useful for individual-level assessments. Indeed, in the domain of hearing and speech processing, several groups have demonstrated robust correlations between neural speech-tracking metrics and speech intelligibility, for example, in children with dyslexia or in those with hearing impairment (Keshavarzi et al., 2022; Xiu et al., 2022; Van Hirtum et al., 2023). In contexts that require selective attention to a target talker, speech-tracking methods have also been successfully applied to distinguish between the neural representations of target and non-target speech in individual participants and even on a per-trial basis (a classification-based approach that can be effective, for example, for designing neuro-steering devices or hearing aids; Henshaw and Ferguson, 2013; O’Sullivan et al., 2015; Kidd, 2017; Fallahnezhad et al., 2023; see Extended Data Fig. 6-1). However, to date fewer studies have looked at the neural-bias index in individual participants, a metric that offers insights not only into the distinction between target and non-target speech but into the modulation of their neural representations as a function of task relevance, which is thought to be a signature of top-down selective attention (Hillyard et al., 1973; Hansen and Hillyard, 1983; Woods et al., 1984; Bidet-Caulet et al., 2007; Manting et al., 2020).

The neural bias in reconstruction accuracies of target and non-target speech for individual participants is shown qualitatively in several previous papers, but statistical analyses focused mostly on the group level (Ding et al., 2012; Ding and Simon, 2012; Fuglsang et al., 2017; Rosenkranz et al., 2021). Kaufman and Zion Golumbic (2023) introduced the use of attention-agnostic permutation tests to quantify and test the neural-bias index statistically in individual listeners. Results from that MEG study are similar to those found here, whereby fewer than half of the participants (∼30% in that study) exhibited significant modulation of speech tracking by selective attention, even when using a “permissive” statistical threshold. The fact that group-level averages consistently show a robust difference between target and non-target speech despite the underlying variability across participants is explained by the asymmetric distribution of the neural-bias metric—since none of the participants show an “opposite” bias (i.e., more accurate reconstruction of non-target vs target speech). This asymmetric distribution also lends further credibility to the neural-bias metric as reliably capturing the relative neural representation of the two speech stimuli.

How should we interpret the variability in neural bias across individuals? Here we ruled out several trivial explanations, namely, that variability is due to poor EEG signal quality, poor speech-tracking abilities, or poor attention to the target—since we show that the reconstruction accuracies for target and non-target speech are correlated with each other, but their average is not correlated with the neural-bias index, nor is it correlated with accuracy in answering questions about the content of target speech. Instead, we offer an alternative—admittedly speculative—interpretation that emphasizes possible variability across individuals in their de facto allocation of processing resources among competing talkers. We know from our subjective experience that paying attention solely to one target speech and shutting out other competing stimuli can be extremely difficult. There are numerous examples that both sensory and semantic properties of non-target speech are encoded and processed, indicating that selective attention to one talker does not imply its exclusive representation (Dupoux et al., 2003; Rivenez et al., 2006; Beaman et al., 2007; Parmentier et al., 2018; Vachon et al., 2019; Har-shai Yahav and Zion Golumbic, 2021; Brown et al., 2023; Har-Shai Yahav et al., 2024). Moreover, the fact that individuals can divide their attention reasonably well between two concurrent speech streams if asked to do so indicates that sufficient perceptual cognitive resources may be available to listeners to apply a multiplexed listening strategy (Vanthornhout et al., 2019; Agmon et al., 2021; Kaufman and Zion Golumbic, 2023). Given that, it is reasonable to assume that even when instructed to pay attention to only one talker, listeners may devote at least some resources to the competing non-target talker as well—either voluntarily, as in divided attention, or involuntarily (Makov et al., 2023). This notion is in line with “load theory of attention,” which suggests that rather than attributing attentional selection of target stimuli/features to either “early” or “late” stages of processing, attention should be viewed as the dynamic allocation of available cognitive resources among competing stimuli. This allocation of resources among talkers reflects their prioritization vis-à-vis their relevance to the listener but can also vary as a function of perceptual load, task demands, and listener motivation/internal state, which we propose may underlie some of the variability observed here between participants (Lavie et al., 2004; Wild et al., 2012; Gagné et al., 2017; Murphy et al., 2017; Peelle, 2018). Along these lines, the lack of a correlation between neural bias and performance may suggest that the perceptual and cognitive demands of the current task, which emulates those encountered in many real-life situations, left many listeners with sufficient available resources to corepresent both talkers without behavioral costs. Clearly, this interpretation is speculative and the current data are insufficient for testing its plausibility; however, we offer it as a hypothesis for future studies aimed at better understanding individual differences in prioritizing between target and non-target speech under realistic circumstances and whether this variability is explained by specific personal traits, by perceptual or cognitive load, or by other state-related factors (Beaman et al., 2007; Colflesh and Conway, 2007; Forster and Lavie, 2008; Sörqvist and Rönnberg, 2014; Murphy et al., 2017; Lambez et al., 2020).

Another important point to note in this regard is that speech tracking, as quantified here as the envelope-following response measured using EEG, captures only a partial neural representation of the speech, primarily reflecting encoding of its acoustic properties in auditory (Ding et al., 2012; Mesgarani and Chang, 2012; Zion Golumbic et al., 2013b; Crosse et al., 2016; Fiedler et al., 2019). Recent work that has attempted to separate between neural tracking of acoustic and linguistic/semantic features of speech has suggested that selective attention primarily affects the latter (Lachter et al., 2004; Ding et al., 2018; Brodbeck et al., 2018a), although this is not always the case (Parmentier, 2008; Parmentier et al., 2018; Vachon et al., 2019; Har-shai Yahav and Zion Golumbic, 2021). Moreover, studies that have looked at neural speech tracking across different brain regions, using source-level MEG data or intracranial EEG (ECoG) recordings, have shown a dissociation between the sensory cortical regions that corepresent concurrent speech versus higher-order regions (e.g., anterior temporal cortex, as well as frontal and parietal regions) where attention selectivity was more prominent (Zion Golumbic et al., 2013b; Brodbeck et al., 2018a,b; Har-shai Yahav and Zion Golumbic, 2021). Accordingly, it is possible that if we were to use a more detailed characterization of the speech stimuli, had used a more complex non-linear model, or had analyzed neural responses stemming from brain regions beyond auditory cortex, we might have found more extensive evidence for neural bias in individual participants. While this is an important limitation to consider from a basic-science perspective, the use of EEG in the current study and our focus on the acoustic envelope of speech have critical applicational value. The motivation to derive personalized neural metrics of selective attention (as opposed to group-based data) has a large practical component, such as providing tools for clinical/educational assessments and interventions. As such, these would likely involve EEG recordings (which are substantially more accessible and affordable than MEG) and—in the case of speech processing—would rely on analyzing speech features that are easy to derive (and do not require a tedious annotation process; Agmon et al., 2023). In publishing this data set and emphasizing the variability across individuals, we hope to provide a transparent account of the complexity of using EEG-based speech-tracking data, as a “biomarker” of selective attention in individual listeners, and emphasize the need to systematically investigate the factors underlying this variability (be them “cognitive” or “methodological” in nature).

### Conclusion

In traditional cognitive neuroscience research, there is a desire to manipulate a specific construct (e.g., which stimulus is the target) while controlling for all low-level sensory differences between stimuli. However, as we turn to studying neural operations under increasingly ecological conditions, perfect control is less possible. In the case of selective attention, “targetness” is often accompanied with specific sensory attributes, making targets inherently different than non-targets. Here we studied one such case—where target speech is audiovisual and non-target speech is peripheral and auditory only. We show that under these more realistic conditions, the hallmark signature of selective attention—namely, the modulation of sensory representation and its robustness to switches in target identity—is conserved, at least at the group level. At the same time, our results also point to the underlying diversity among participants in how that this modulation manifests, to the degree that in over half of the participants target and non-target speech were represented just as well (albeit in different ways). These results emphasize that there is still much to explore regarding how the brain—or how different brains—treats target and non-target speech when attempting to achieve selective attention. This work calls for more granular investigation of how factors such as task difficulty, perceptual load, listener motivation, and personal traits ultimately affect neural encoding of competing stimuli, under ecological conditions.

## Synthesis

Reviewing Editor: Catherine Schevon, Columbia University Decisions are customarily a result of the Reviewing Editor and the peer reviewers coming together and discussing their recommendations until a consensus is reached. When revisions are invited, a fact-based synthesis statement explaining their decision and outlining what is needed to prepare a revision will be listed below. The following reviewer(s) agreed to reveal their identity: NONE.

Both reviewers provided several critical points regarding the analysis strategy and the interpretation of the results. They need to be carefully considered in a revision. Since both reviewers provide detailed comments, I here provide all comments of the reviewers to allow the authors to reply point-by-point to these comments.

Reviewer 1:

This manuscript presents an investigation on how attention switching impacts the neural encoding of speech in a two-talker environment. The rationale was that switching attention might impact the re-engagement in various ways, from an improved suppression of the unattended stream due to the increased familiarity with the speaker, to an increased difficulty in performing the selective attention task, due to the a higher distractibility towards the formerly-attended stream.

The idea and data are interesting. However, there are some fundamental flaws in some of the analyses and discussion that mine the validity of the results, if not addressed.

Please find my comments below. Please note that line numbers are missing, hence my comments cannot point to the specific lines.

Major comments

1. The main dependent measure seems to be the comparison between the prediction correlations for the target model and the non-target model. While that effect was found in previous work, it is not the most important one. In fact, markers of the segregation of target and non-target (rather than overall encoding) are more sensitive to selective attention, and that in fact what is also used for decoding tasks (e.g., O'Sullivan et al., Cereb. Cortex, 2014). Typically, such markers are derived by fitting a target decoding model, and then by correlating the reconstructed envelope to the two possible envelopes. The resulting classification scores (or d-prime) quantify the ability to filter EEG activity reflecting the target speech more than the non-target. Why did the authors use such a suboptimal metric, when a stronger and more direct measurement was available? Please note that comparing target and non-target models that are fit separately assumes that the brain encoding of the target is stronger than for the non-target speech. Given the goals of this study, that is an unnecessary assumption, as the goal should be to determine if target and non-target are segregated (while the weaker encoding would be a byproduct of that segregation).

2. The authors have decided to optimise data from all participants as a group, selecting a common lambda value. While one might have some reasons to do so with encoding models (e.g., Crosse et al., Front. in Hum Neurosci, 2021), I don't see a reason for doing that with decoding models. In fact, that would lead to suboptimal results by definition, while the point of a decoding model is to get the best possible decoding (rather than interpreting the weights). This choise is particularly problematic on the claim of the individual subject differences. So, I find this choice to be in contradiction with the intent of looking into the individual participants. That should be done by considering the participants independently, with completely independent models, instead of using a "group-level" choice of the lambda.

3. The study does not control (as far as I could see) for potential effect of fatigue. That effect could be excluded by including a control condition with the same duration as the typical trials, but without a switch. That is a confound that might mask interesting effects. That issue was timidly mentioned in the discussion, while I think it is a main issue that would have been easy to address. Could the authors comment on that or potentially collect additional data?

4. On that same analysis (before vs. after): There are other issues that hamper the ability to claim anything on that point. Indeed, lack of a significance difference doesn't mean that there is no difference. But my main issue with that n.s. effect is that the authors do not seem to be really trying to get an effect there. First, a stronger metric should be used (see major comment 1). Second, the re-use of the same lecture as speech material would likely reduce any distraction effect of the non-target, as that would be somewhat familiar even in the "before" condition. So, in my view, the current data cannot support that claim due to these several issues.

5. The authors claim (in multiple pars of the paper) that a strong or significant envelope tracking "rules out" the possibility that a low EEG signal quality could explain why most participants did not show target > non-target. One of the factors that might impact that was discussed in the first and second major points. But there is a more general consideration that is crucial here. Envelope tracking measurements can reflect a number of neural sources and processes, and only few of them might reflect the separation between target and non-target. So, good envelope tracking might mean that the EEG signal is capturing some acoustic- or speech-sensitive neural sources. But it doesn't imply that it captures signals that are sensitive to selective attention. So that argument does not hold.

6. Page 15 (middle paragraph). One of the reasons for looking at EEG results at the group level is to determine if an effect is consistent in a statistical sense. That is often because of the low SNR of EEG signals and consequent large variability across participants. The authors found effects that are significant at the group level, with large variability at the single subject level. They then claim that such a variability reflects different neural encodings or different behaviour across participants. That might be true, but another even more likely explanation is that the data is just too little and too noisy to fit reliable single subject models. This is particularly true in this study, which used less data than in previous work, from what I could see (30 min per participant; so, 15 min per condition?). This is an important issue as it hampers one of the key claims of the study. One suggestion is to run the same analysis on publicly available data (e.g., O'Sullivan, which used ~1 hour of data I think) and to measure how many participants show significance when using 15 min of data, 30 min, 45 min, and 60 min. Would it be reasonable to think that 25% of the participants would show significance with 15 min of data, and that the percentage would increase when using more data? That, or some other control, is necessary to provide some support to the author's claim.

Other comments

1. There are several unmotivated experimental choices that appear to be afterthoughts. For example, why was the non-target presented only on the left? What not left and right, maybe in alternation or simultaneously? Why did the authors decide to use an AV target. How does the speech envelope correlate with the visual motion? Could part of the target vs. non-target effect be explained by the presence of the V component, or is that completely uncorrelated to the envelope?

2. It seems that the EEG data was filtered twice. First between 0.5 and 40 Hz, then between 1 and 20 Hz. Could the authors elaborate on that point? Also, what filters were used (type and order)? Were they zero-phase shift? Filtering is a delicate operation that can damage the data.

3. Figure 3A: It would be useful to see that result before and after the switch, and for target and non-target, as in the other panels

4. It wasn't clear to me how the encoding and decoding models were fit exactly? Were separate models fit for before and after? That would lead to even smaller training data, which would be problematic. What about Target and Non-Target?

5. It seems that the z-score thresholds were picked arbitrarily, with little justification. Could the authors elaborate on that? That looks important, as a different threshold would change that 25%.

6. Is the data going to be publicly available? I strongly recommend that.

Reviewer 2:

The paper presents an experiment investigating the neural tracking of speech signals in the presence of a competing, differently localised speaker. The target speaker is always presented in front of the listener, with a supporting video and the competing speaker is presented to the left. The authors use a TRF approach to estimate the neural tracking of both speakers and further use the difference in (envelope) reconstruction accuracy for target and ignored talker as an index of attention bias. They report that attention bias (calculated in this way) is at best a poor predictor of behaviour, varying within substantially between participants and within participant over experimental segments.

This is in many ways an intriguing report, but there are some issues that, in my opinion, reduce the confidence we may have in the results as they are currently presented and interpreted.

The paper is overall well written and presented, the introduction covers a satisfactory range of relevant recent work and frames the work adequately (although note comment below relating to alpha-band power). The authors raise an important point regarding the significance of individual differences. However, there are methodological details that are at times insufficiently clearly described and choices pertaining to the experimental design and analyses are in part not sufficiently explained.

In particular, it is unclear how the authors conceive of "attention switch". I believe the paradigm can be used to probe sustained (selective) attention to only one target (the speaker presented with video) in the presence of a lateralised distractor. I found the construct of switching to be over-emphasised, given that there is only one switch of talker, which did not entail a change in locus or modality of attention in order to continue effectively executing the task, and that, furthermore, the trial in which this switch occurs is explicitly not analysed.

I am also somewhat concerned about a circularity of argumentation. It appears that attention is defined as a function of a specific neural corelate (i.e. the attention bias, derived from the TRF). Is this a valid definition of attention? How confident can we be that the measure of attention bias is a measure of attention? It may well be an index that is affected by attention, but given that it is not well predictive of behavioural outcomes, there is an implicit implication that auditory selective attention must therefore not be relevant to behavioural performance in this task. I do not think this is the message the authors are trying to convey, thus caution is required in describing the nature of the effects.

In sum, the paper presents an interesting study with an intriguing outcome, but the core cognitive construct requires more comprehensive elucidation and the design and analysis require additional explanation and justification.

Main Concerns:

The description of the loudspeaker position follows the description of the presentation of the distractor to the left. In order to make it clear where the target is presented, specify the location of loudspeakers first.

An analysis of audio from on-ear headphones is mentioned - if the listener is concurrently receiving auditory input from headphones alongside the free field, this must be discussed, and any potential interference must be accounted for. Wearing on-ear headphones will have at least one consequence that is of particular relevance in the context of sound localisation - it will eliminate cues for localisation that are generated by the transfer functions of the pinnae. This appears to have the unfortunate consequence of reducing the ecological validity of the free-field auditory presentation.

Justification for the target speaker being presented with video. It is known that neural tracking of speech signals increases (i.e. is altered) in the presence of concurrent visual input. This is briefly mentioned in the discussion, but the potential consequences of one speech stream being supported by visual input and the other not is potentially greater than merely increasing the amplitude of entrainment. The role of, for example, the motor cortex (e.g. Park, Kayser, Thut &Gross, 2016)) in mediating auditory entrainment to the speech signal may have consequences for the response function that relates the acoustic and cerebral signals.

This brings me on to a further methodological question: Is TRF the correct approach for assessing neural tracking? The measure provides an insight into the stability of the linear relationship between R and S, but it is not clear to what extent they truly reflect tracking of the envelope. Can the authors dedicate some effort to explaining why they favoured this specific method over others (such as phase-locking index, Gaussian Copula Mutual Information, etc.)? It would also be valuable to know more about the justification for the broadband speech envelope employed.

Other work has focused on lateralised changes to the oscillatory cerebral signal (particularly in the alpha band) as a function of lateralised auditory attention (Wöstmann and colleagues, e.g. Wöstmann, Alavash &Obleser, 2019; Wöstmann, Lim &Obleser 2017). There is no mention of this research, which seems like an oversight when summarising the current state of the field, even if the TRF method is not directly comparable to time-frequency analysis.

Minor points and requests for clarification:

Specify the interpolation method applied, describe how cardiac artefacts were identified

Please clarify or homogenise: "Trials" and "Sentences"

Please provide the reference for "Liberman's cochlear frequency map"

Provide more detail on the calculation of Bayes factors - report the priors in sufficient detail to allow future replication of the analyses, even if the defaults of the software should change, or in other software.

## Author Response

We greatly appreciate the reviewers' feedback and comments on our manuscript, and have made substantial modification to the text and to some of the analyses, to address their concerns. The current version focuses more on the basic effect of selective attention, emphasizing the spatial-realism and impact of using audiovisual speech, as well as the differences between averaging data across the sample vs. recognizing the variability across individuals. As part of our revision, we have also changed the title of the paper to: "Neural Speech-Tracking During Selective Attention: A Spatially Realistic Audiovisual Study", and have added some Extended Data to address Reviewer #1's concern about the decoding approach used here.

Below, please find a detailed response in purple to the specific points raised by the reviewers.

Reviewer 1:

This manuscript presents an investigation on how attention switching impacts the neural encoding of speech in a two-talker environment. The rationale was that switching attention might impact the re-engagement in various ways, from an improved suppression of the unattended stream due to the increased familiarity with the speaker, to an increased difficulty in performing the selective attention task, due to the a higher distractibility towards the formerly-attended stream.

The idea and data are interesting. However, there are some fundamental flaws in some of the analyses and discussion that mine the validity of the results, if not addressed.

Please find my comments below. Please note that line numbers are missing, hence my comments cannot point to the specific lines.

Major comments 1. The main dependent measure seems to be the comparison between the prediction correlations for the target model and the non-target model. While that effect was found in previous work, it is not the most important one. In fact, markers of the segregation of target and non-target (rather than overall encoding) are more sensitive to selective attention, and that in fact what is also used for decoding tasks (e.g., O'Sullivan et al., Cereb. Cortex, 2014). Typically, such markers are derived by fitting a target decoding model, and then by correlating the reconstructed envelope to the two possible envelopes. The resulting classification scores (or d-prime) quantify the ability to filter EEG activity reflecting the target speech more than the non-target. Why did the authors use such a suboptimal metric, when a stronger and more direct measurement was available? We are aware of the classification method used by O'Sullivan et al. and others, in which the goal is to identify which of two competing stimuli was the "target" speech in a given trial, based on a model trained specifically on "target" speech (and vice-versa for the "non-target" speech).

This approach does indeed differ in its rationale from the one used here, where we train two separate models on "target" and "non-target" speech (using a multivariate approach), and compare the 'goodness of fit' (predictive power) of each model.

There are advantages and disadvantages to the two approaches, and both have been used extensively in studies of selective attention to speech (Fuglsang et al., 2017; Har-shai Yahav et al., 2023; Kaufman &Zion Golumbic, 2023; Mirkovic et al., 2015; A. E. O'Sullivan et al., 2019; J. A. O'Sullivan et al., 2015; Teoh &Lalor, 2019). Rather than stating that one approach is 'superior', 'optimal' or 'more sensitive' than the other, we believe that they serve different scientific purposes and that the classification approach suggested by the reviewer would not be appropriate for addressing the scientific questions posed here. We explain the rationale for this choice below:

1) The classification approach can be useful if we want to ask: is it possible to distinguish between the neural representation of target and non-target speech. For this purpose, demonstrating that a decoder trained on target speech can decode target speech better than non-target speech would indicate that their neural representations are "not the same". Accordingly, (as also shown in original paper by O'Sullivan et al.), the same pattern of results could be obtained when a decoder is trained on non-target speech and tested on target speech - here too, the decoder would not perform as well. Successful classification - while potentially useful for engineering purposes, such as controlling a neuro-steered hearing aid or other device - can be easily explained by differences in the acoustic, spatial and other perceptual characteristics of target and non-target speech (and in our case, by the fact that target-speech was audiovisual), which would undoubtedly lead to finding decoders with different spatio-temporal characteristics for each stimulus. This is seen nicely when using encoding models - the TRFs derived for target and non-target speech differ in many spatio-temporal properties, so it is not surprising that they would not be successful if used to predict stimuli from the opposite class. Accordingly - successful classification in and of itself should not be interpreted as a "pure" effect of selective attention, as it is heavily influenced by the differences between the stimuli themselves.

2) The scientific question we ask here is slightly different. We want to know whether target speech is represented more robustly in the neural data than non-target speech, a pattern that is considered a signature of 'selective attention' - i.e., enhancement of target speech and/or suppression of non-target speech (Ding et al., 2012; Fiedler et al., 2019; Kerlin et al., 2010; O'Sullivan et al., 2015; Zion Golumbic, Ding, et al., 2013). For this purpose, we believe that is it more appropriate to optimize decoders for each stimulus separately (thus accounting for their differences in properties), and then assess how well each one performs for predicting the stimulus it was trained on (the model's goodness-of-fit/predictive power/accuracy). Using this approach, if we find that both decoders perform very well - this indicates that both stimuli are represented with similar precision in the neural response. Conversely, finding that the decoder for one stimulus outperforms the other can be interpreted as superior or more detailed neural encoding of that stimulus relative to the other, effects that have been associated with better intelligibly and/or higher levels of attention to this stimulus (Best et al., 2008; Getzmann et al., 2017; Lin &Carlile, 2015; Orf et al., 2023; Teoh &Lalor, 2019; Uhrig et al., 2022) .

We have clarified this rationale in the revised manuscript (Methods p. 10; and Discussion p. 23). In addition, we have added an Extended Data analysis (Figure S1) where we demonstrate the results obtained using the current approach vs. the classification approach suggested by the reviewer. As the reviewer anticipated, using a decoder trained on one class of stimuli (target/non-target) to distinguish between the two classes is indeed more 'sensitive', however, in our opinion does not teach us much about selective attention - just that these are different stimuli. We hope that by articulating the differences between these two approaches and the inferences that can be derived from each one, our work will contribute to formulating best-practices for using speech-tracking methods to study aspects of selective attention.

Please note that comparing target and non-target models that are fit separately assumes that the brain encoding of the target is stronger than for the non-target speech. Given the goals of this study, that is an unnecessary assumption, as the goal should be to determine if target and non-target are segregated (while the weaker encoding would be a byproduct of that segregation).

Perhaps we misunderstood the reviewer's intention here, but - to clarify - the multivariate model used to train the decoders for target and non-target speech makes no assumption regarding any difference between them. The model is agnostic to any 'sensory/cognitive distinction' between the two stimuli, therefore any differences that emerge in the 'goodness of fit' of the two decoders, can reliably be attributed to differences in their neural coding.

Regarding the goals of the study - from our perspective, this is exactly what we wanted to study: whether there is weaker encoding for non-target speech relative to target speech, and whether this effect is maintained when the role of the two talkers is switched. Whether the two decoders are different (and therefore "segregated" is trivial in our opinion, given the difference in their spatial and audiovisual properties - as elaborated in response to the previous comment).

2. The authors have decided to optimise data from all participants as a group, selecting a common lambda value. While one might have some reasons to do so with encoding models (e.g., Crosse et al., Front. in Hum Neurosci, 2021), I don't see a reason for doing that with decoding models. In fact, that would lead to suboptimal results by definition, while the point of a decoding model is to get the best possible decoding (rather than interpreting the weights). This choise is particularly problematic on the claim of the individual subject differences. So, I find this choice to be in contradiction with the intent of looking into the individual participants. That should be done by considering the participants independently, with completely independent models, instead of using a "group-level" choice of the lambda.

Thank you for this comment. We have re-run the decoding analyses and chose the most 'optimal' lambda for each participant. However, in practice in 21/23 participants the best lambda was the same (lambda = 1000) and for the two participants where the optimal lambda was different (lambda = 500), the difference in r-values between the two lambdas was <0.005. Therefore, the use of a group-level lambda here is indistinguishable from selecting personal lambdas.

We have clarified this in the text.

3. The study does not control (as far as I could see) for potential effect of fatigue. That effect could be excluded by including a control condition with the same duration as the typical trials, but without a switch. That is a confound that might mask interesting effects. That issue was timidly mentioned in the discussion, while I think it is a main issue that would have been easy to address. Could the authors comment on that or potentially collect additional data? Indeed, the issue of fatigue is extremely interesting in the context of selective attention. We agree that if we had found reduced speech tracking/neural bias in the 2nd half of the experiment, this could have been confounded with fatigue. However, since here we did not find any difference between the 1st vs. 2nd half of the experiment, this suggests that fatigue probably did not play a big role here and supports the claim that speech tracking and neural-bias remains robust over time despite the switch in talkers. Unfortunately, in the current design the data is insufficient for reliable comparison of results in smaller portions of the experiment (e.g., in each quarter).

It is quite possible that switching the target-talker mid-way could have mitigated effects of fatigue that otherwise would have occurred, but this is a question for future studies. We now address the issue of fatigue in Discussion p. 22).

4. On that same analysis (before vs. after): There are other issues that hamper the ability to claim anything on that point. Indeed, lack of a significance difference doesn't mean that there is no difference. But my main issue with that n.s. effect is that the authors do not seem to be really trying to get an effect there. First, a stronger metric should be used (see major comment 1). Second, the re-use of the same lecture as speech material would likely reduce any distraction effect of the non-target, as that would be somewhat familiar even in the "before" condition. So, in my view, the current data cannot support that claim due to these several issues.

We fully agree that caution needs to be exercised not to over-interpret null-results, and have edited the text to reflect this (Discussion p. 22).

To the reviewer's point regarding the use of a 'stronger metric' please see our response to comment #1, where we argue that the alternative metric suggested would in fact be uninformative (relative to the research questions asked here), given the use of spatial and audiovisual speech in the current experimental setup.

To the reviewer's comment regarding the "re-use of the same lecture" we would like to clarify that none of the speech materials were repeated in the experiment. Rather, we used different segments of the same lecture in part 1 and part 2 of the experiment. We have now clarified this in the Methods section (p. 6) To the broader concern regarding the reliability of difference/lack-thereof between the first and second half of the experiment, in the revised manuscript we now include additional statistical analyses to address this point: • In the group-analysis, we have added Bayes analysis of the difference between the two parts, indicating that the null result is supported (p.14). "An ANOVA comparing the reconstruction accuracies for target vs. non-target speech across both halves of the experiment, revealed a main effect of task-relevance [target vs. non-target: F(1,22)=28.3, p<0.001] but no main effect of half [F(1,22)=0.12, p=0.73] or interaction between the factors [half x talker: F(1,22)=0.87, p=0.87)]. These results were confirmed using a Bayesian ANOVA which indicated that the main effect of task-relevance could be embraced with high confidence and explains most of the variance (BFinclusion = 317, p=0.002) but there was no reliable difference between the two halves or interaction (BFinclusion = 0.26 and BFinclusion = 0.27 respectively, both ps > 0.7). • In the individual-level analysis, we have added permutations tests assessing the chance-level of finding changes in decoding accuracy and/or changes in the size/direction of neural-bias between the two parts. This analysis also shows a lack of difference between the two halves even in individual participants (where the speech-materials were actually different in the two halves; Methods p.9, Figure 2; Results p.16): "We performed a third permutation test to assess whether speech reconstruction accuracies and the Neural-Bias index differed significantly in the two halves of the experiment, i.e., before vs. after the talker-switch. For this, we conducted an "order-agnostic" permutation test where trials were randomly re-labeled so that the data included in each regressor were 50% from the first half of the experiment and 50% from the second half (Figure 2C). Multivariate decoders were trained on this re-labeled data and reconstruction accuracy values were estimated for each regressor and this procedure was repeated 100 times, yielding a null-distribution. A participant was considered as showing a significant difference in neural-tracking of either the target or non-target talker, or different in neural-bias, if the their real-data fell in the top 5%tile of the relevant null-distribution." We have also added a cross-over test, whereby we assess how well decoders trained on target/non-target speech in one part of the experiment, are able to predict stimuli from the other part of the experiment (Figure 5). This analysis showed that decoders trained in one half of the experiment generalized well to the other half when tested on stimuli that shared the same task-relevance (role), but did not generalize well to stimuli that shared the same talker-identity. This suggests that the decoders are largely invariant to talker-identity, and primarily capture features related to the role of the talker in the given task (in this case, being the target talker, presented audio-visually from a central location).

5. The authors claim (in multiple parts of the paper) that a strong or significant envelope tracking "rules out" the possibility that a low EEG signal quality could explain why most participants did not show target > non-target. One of the factors that might impact that was discussed in the first and second major points. But there is a more general consideration that is crucial here. Envelope tracking measurements can reflect a number of neural sources and processes, and only few of them might reflect the separation between target and non-target. So, good envelope tracking might mean that the EEG signal is capturing some acoustic- or speech-sensitive neural sources. But it doesn't imply that it captures signals that are sensitive to selective attention. So that argument does not hold.

We fully agree with the reviewer that measures of envelope-tracking represent only one, out of many potential mechanisms that the brain likely engages for speech processing, source separation etc. We have toned-down our language in multiple places throughout the manuscript, to clarify that the current results pertain only to this specific measure, which limits more general claims about attention.

See for example Discussion p.26: "Another important point to note in this regard is that speech-tracking as quantified here as the envelope-following response measured using EEG, captures only a partial neural representation of the speech, primarily reflecting encoding of its acoustic properties in auditory".

6. Page 15 (middle paragraph). One of the reasons for looking at EEG results at the group level is to determine if an effect is consistent in a statistical sense. That is often because of the low SNR of EEG signals and consequent large variability across participants. The authors found effects that are significant at the group level, with large variability at the single subject level. They then claim that such a variability reflects different neural encodings or different behaviour across participants. That might be true, but another even more likely explanation is that the data is just too little and too noisy to fit reliable single subject models. This is particularly true in this study, which used less data than in previous work, from what I could see (30 min per participant; so, 15 min per condition?). This is an important issue as it hampers one of the key claims of the study.

One suggestion is to run the same analysis on publicly available data (e.g., O'Sullivan, which used ~1 hour of data I think) and to measure how many participants show significance when using 15 min of data, 30 min, 45 min, and 60 min. Would it be reasonable to think that 25% of the participants would show significance with 15 min of data, and that the percentage would increase when using more data? That, or some other control, is necessary to provide some support to the author's claim.

We agree that the amount of data can critically affect the reliability of results, particularly when working with single-subject data.

In a recent paper, Mesik and Wojtczak (2023) tested how the amounts of training data affects the validity of TRF models, and found that approximately 8 minutes of clean data should be sufficient for estimating reliable TRFs in most participants. However, since in that work only single-speaker data was used, we have now conducted several additional analyses on the current data set to estimate the expected chance-level given the amount of data collected in the current design (~30 minutes total, 15 minutes per half). Specifically, we now report the results of within-participant permutations tests looking at the reliability of a) the decoding accuracy for each speaker (in the full experiment and in each half); b) the difference in decoding between target and non-target speech (in the full experiment and in each half); and c) the difference in decoding between the first and second half of the experiment. These analyses are described below and are now included in the revised manuscript (see new Figure 2 for an illustration of these analyses, pasted here).

New Figure 2. Data-driven permutation tests for individual-level statistics. Three permutation tests were designed to assess statistical significance of different results in individual participants. The black rectangles in all panels show the original data organization on the left and the re-labeling for permutations test on the right. A. S-R permutation test. In each permutation, the pairing between acoustic envelopes (S) and neural data responses (R) was shuffled across trials such that speech-envelopes presented in one trial (both target &non-target speech) were paired with the neural response (R) from in a different trial. This yields a null-distribution of reconstruction accuracies that could be obtained by chance, to which the real-data can be compared (right). B. Attention-agnostic permutation test. In each permutation, the target and non-target speech stimuli were randomly re-labeled to create attention-agnostic regressors that contain 50% target speech and 50% non-target speech. The reconstruction accuracy for each regressor was estimated and the difference between them is used to create a null-distribution to which the neural-bias index can be compared (right). C. Order-agnostic permutation test. In each permutation, trials were randomly re-labeled and separated into two order-agnostic groups consisting of 50% trials from the first half of the experiment and 50% trials from the second half. The reconstruction accuracy for each group of trials was estimated and the difference between them is used to create a null-distribution to which the real data from each half of the experiment can be compared (right).

We are also making this data set publicly available, so that other researchers can test our methods or use them to develop alternative approaches. Results from these analyses demonstrate the following: a) As shown in the new Figure 6, when analyzing data from the entire experiment, 22 out of 23 participants showed significant decoding values for at least one of the speech-stimuli. This provides data-driven evidence for the robustness of the speech-tracking metric in individual participants, despite the noisier nature of single-subject EEG. When we compare the average decoding accuracy to the neural-bias index in individual participants (revised Figure 6, pasted below), we find no correlation between them. In other words, even participants who had very good decoding accuracies, did not necessarily show large neural-bias for target speech. This result is in line with the findings reported by Kaufmann &Zion Golumbic (2022), and in our opinion support the claim that the lack of a significant neural-bias cannot be "trivially" explained by poor data quality. We now clarify this rationale in the Discussion as well (p. 25) Revised Figure 6. Speech Reconstruction and Neural-bias in individual participants - Full experiment. A. Left: Bar graphs depicting reconstruction accuracy in individual participants for target (black) and non-target (dark gray) speech. Horizontal light gray lines represent the p=0.05 chance-level, derived for each participant based on data-driven S-R permutation. Asterisks indicate participants who also showed significant neural-bias to target speech (see panel B). Right: Scatter plot showing reconstruction accuracies for target and non-target speech across all participants. The red line represents the linear regression fit between the two variables, which was significant (Pearson's r = 0.43, p = 0.038). B. Scatter plot showing the average reconstruction accuracy and neural-bias index across participants, which were not significantly correlated. Vertical dashed lines indicate the threshold for significant neural-bias (z=1.64, one-tailed; p<0.05). C. Scatter plots showing the accuracy on behavioral task vs. reconstruction accuracy of target speech (left), non-target speech (middle) and the neural-bias index (right), across all participants. No significant correlations were found. b) We also repeated these same analyses on each half of the experiment separately. As the reviewer anticipated, chance-levels were higher when using only half of the data (range between r = 0.02-0.03) relative to when using the full data set (range between r = 0.015-0.02). Nonetheless, significant decoding (of at least one talker) was still found in the vast majority of participants (19/23 in each part, but not necessarily the same participants; see Figures below), even if this was not as ubiquitous as when using all the data.

Here too there was no correspondence between decoding accuracy and the neural-bias index, and further weakening the claim that a lack of neural-bias is due to poor EEG quality. We hope that by conducting data-driven statistical analyses in individual participants and transparently presenting these data, our results will contribute to ongoing efforts to optimize the means for obtaining reliable and interpretable speech tracking data from individuals (including the amount of data needed).

Panel A from revised Figures 7&8 Bar graphs depicting reconstruction accuracy in individual participants for target (black) and non-target (dark gray) speech, in each half of the experiment separately. Horizontal light gray lines represent the p=0.05 chance-level, derived for each participant based on data-driven S-R permutation.

Other comments 1. There are several unmotivated experimental choices that appear to be afterthoughts. For example, why was the non-target presented only on the left? What not left and right, maybe in alternation or simultaneously? We are happy to clarify. This choice of presented non-target speech from one side (left) stemmed purely out of our desire to maximize amount of data in each condition, and avoid the need to double the length of the experiment to accommodate two presentation locations. We chose to keep this location constant for all participants since we assumed that presenting speech from different spatial locations might result in different TRF filters, which would make it even more difficult to compare results between participants. However, we fully agree that future studies should be designed looking specifically into the effect of spatial location per se.

We did not want to use 'simultaneous' presentation to two-sides, as this would invalidate the spatial-realism of the setup.

We have now clarified this in the text (p.7) Why did the authors decide to use an AV target. How does the speech envelope correlate with the visual motion? Could part of the target vs. non-target effect be explained by the presence of the V component, or is that completely uncorrelated to the envelope? This is another excellent point. Yes, it is highly likely that the visual input contributed to the better tracking of the AV movie, as have been shown in several previous studies (Ahmed, Nidiffer, &Lalor, 2023; Ahmed, Nidiffer, O'Sullivan, et al., 2023; Crosse et al., 2015; Fleming et al., 2021; Fu et al., 2019; Grant &Seitz, 2000; Haider et al., 2024; Karthik et al., 2024; Schwartz et al., 2004; Sumby &Pollack, 1954; Wikman et al., 2024; Zion Golumbic, Cogan, et al., 2013). And yes, looking at the person you are meant to pay attention to IS expected to explain part of the increase in speech-tracking for target speech, although it is impossible to separate the contribution of the "visual motion" from the contribution of "attention".

The choice to use a movie as the target stimulus was motivated by the aspiration to study selective attention to speech under more ecological and relevant contexts. Specifically, this study simulates the case where individuals are asked to pay attention to an actual person speaking, rather than simply a voice without a body. Eventhough this introduces yet another difference bewteen the target and non-target speech, this is closer to what the brain encounters in real-life - where the listener looks at a target speaker, but still may hear audio stemming from other irrelevant locations.

We now discuss this point more in depth in the Introduction (p.3) and Discussion (p.22).

2. It seems that the EEG data was filtered twice. First between 0.5 and 40 Hz, then between 1 and 20 Hz. Could the authors elaborate on that point? Also, what filters were used (type and order)? Were they zero-phase shift? Filtering is a delicate operation that can damage the data.

Yes, the data was indeed filtered twice. The first filter is done as part of the preprocessing procedure, in order to remove extremely high- and low- frequency noise but retain most activity within the range of "neural" activity. This broad range is mostly important for the ICA procedure, so as to adequately separate between ocular and neural contribution to the recorded signal.

However, since the speech tracking responses itself is less broadband and is comprised mostly of frequencies present in the speech-envelope itself (mostly <10Hz), a second narrower filter was applied to improve TRF model-fit.

In both cases we used a 4th order zero-phase Butterworth IIR filter with 1-seconds of padding, as implemented in the Fieldtrip toolbox. We have added these details to the manuscript.

3. Figure 3A: It would be useful to see that result before and after the switch, and for target and non-target, as in the other panels For this, please see revised Figure 4 (previously 3) and the new Figure 5.

4. It wasn't clear to me how the encoding and decoding models were fit exactly? Were separate models fit for before and after? That would lead to even smaller training data, which would be problematic. What about Target and Non-Target? We have thoroughly revised the methods section to clarify exactly how models were fit. Indeed, for the analysis comparing the 1st vs. 2nd half of the experiment, separately models were fit for each half, and compared to each other. We have also added a new cross-over analysis, where models trained on data from one half of the experiment were tested on data from the other half (see revised Figure 5). As noted in response to previous comments, we have also added new analyses to assess the reliability of these models, despite being trained on half the amount of data.

5. It seems that the z-score thresholds were picked arbitrarily, with little justification. Could the authors elaborate on that? That looks important, as a different threshold would change that 25%.

Yes, we agree that z-score thresholds can be arbitrary, eventhough they are statistically interpretable in terms of associated p-values. We now report z-values as a continuum (see panel B in revised Figures 6-8), and report the proportion of participants with a significant neural-bias index using both a threshold of z=1.64 (p<0.05 one-tailed) as well as with a more conservative z=1.

6. Is the data going to be publicly available? I strongly recommend that.

Yes, we are preparing the data and will make it publicly available once the paper is published.

Reviewer 2:

The paper presents an experiment investigating the neural tracking of speech signals in the presence of a competing, differently localised speaker. The target speaker is always presented in front of the listener, with a supporting video and the competing speaker is presented to the left. The authors use a TRF approach to estimate the neural tracking of both speakers and further use the difference in (envelope) reconstruction accuracy for target and ignored talker as an index of attention bias. They report that attention bias (calculated in this way) is at best a poor predictor of behaviour, varying within substantially between participants and within participant over experimental segments.

This is in many ways an intriguing report, but there are some issues that, in my opinion, reduce the confidence we may have in the results as they are currently presented and interpreted.

The paper is overall well written and presented, the introduction covers a satisfactory range of relevant recent work and frames the work adequately (although note comment below relating to alpha-band power). The authors raise an important point regarding the significance of individual differences. However, there are methodological details that are at times insufficiently clearly described and choices pertaining to the experimental design and analyses are in part not sufficiently explained.

In particular, it is unclear how the authors conceive of "attention switch". I believe the paradigm can be used to probe sustained (selective) attention to only one target (the speaker presented with video) in the presence of a lateralised distractor. I found the construct of switching to be over-emphasised, given that there is only one switch of talker, which did not entail a change in locus or modality of attention in order to continue effectively executing the task, and that, furthermore, the trial in which this switch occurs is explicitly not analysed.

I am also somewhat concerned about a circularity of argumentation. It appears that attention is defined as a function of a specific neural corelate (i.e. the attention bias, derived from the TRF). Is this a valid definition of attention? How confident can we be that the measure of attention bias is a measure of attention? It may well be an index that is affected by attention, but given that it is not well predictive of behavioural outcomes, there is an implicit implication that auditory selective attention must therefore not be relevant to behavioural performance in this task. I do not think this is the message the authors are trying to convey, thus caution is required in describing the nature of the effects.

In sum, the paper presents an interesting study with an intriguing outcome, but the core cognitive construct requires more comprehensive elucidation and the design and analysis require additional explanation and justification.

Main Concerns:

The description of the loudspeaker position follows the description of the presentation of the distractor to the left. In order to make it clear where the target is presented, specify the location of loudspeakers first.

Thank you, we clarified this in the text (p.6).

An analysis of audio from on-ear headphones is mentioned - if the listener is concurrently receiving auditory input from headphones alongside the free field, this must be discussed, and any potential interference must be accounted for. Wearing on-ear headphones will have at least one consequence that is of particular relevance in the context of sound localisation - it will eliminate cues for localisation that are generated by the transfer functions of the pinnae. This appears to have the unfortunate consequence of reducing the ecological validity of the free-field auditory presentation.

We apologize for our mistake - participants were wearing on-ear microphones, not headphone. Audio was presented free field. (the microphones were used to test if we could segregate the audio of the two speakers without access to the 'clean audio', an analysis that was successful but is orthogonal to the current manuscript.) Justification for the target speaker being presented with video. It is known that neural tracking of speech signals increases (i.e. is altered) in the presence of concurrent visual input. This is briefly mentioned in the discussion, but the potential consequences of one speech stream being supported by visual input and the other not is potentially greater than merely increasing the amplitude of entrainment. The role of, for example, the motor cortex (e.g. Park, Kayser, Thut &Gross, 2016)) in mediating auditory entrainment to the speech signal may have consequences for the response function that relates the acoustic and cerebral signals.

Thank you for highlighting this point. Yes, the AV presentation is a key factor (see response to Rev #1 as well). Visual input likely contributed to the enhanced tracking of the AV movie, contributed by both the visual motion and selective attention. While this creates a difference between target and non-target speech, the decision to use an AV movie was driven by the goal of simulating more ecologically valid listening conditions.

We now discuss this point more in depth in the Introduction (p.3) and Discussion (p.21): "It is likely that differences in spatio-temporal characteristics of TRFs for target and non-target speech are affected both by the specific perceptual attributes of the stimuli themselves (e.g., audiovisual vs. audio presentation, spatial location) as well as by their task-related role (target vs. non-target). In the spatially-realistic experimental design used here, these factors are inherently confounded, just as they are under real-life conditions in which listeners look at the talker that they are trying to pay attention to. [....] Rather than trying to assert whether the differences in TRF are due to "selective attention per se" or to "perceptual differences", we accept that in real-life these often go together. We posit that as selective attention research progresses to more ecologically valid contexts, these factors cannot (and perhaps need not!) be teased apart, but rather should be considered as making inherently joint contributions to the recorded neural signal." This brings me on to a further methodological question: Is TRF the correct approach for assessing neural tracking? The measure provides an insight into the stability of the linear relationship between R and S, but it is not clear to what extent they truly reflect tracking of the envelope. Can the authors dedicate some effort to explaining why they favoured this specific method over others (such as phase-locking index, Gaussian Copula Mutual Information, etc.)? It would also be valuable to know more about the justification for the broadband speech envelope employed.

This is a great question, and one that the speech-tracking community has been actively looking into. Although we agree that the linear nature of the TRF might not fully capture all aspects of the neural response to speech, has been shown in countless studies (in humans and animal models) to be an effective, reliable and simple way to capture basic features of the neural representation of speech and other dynamic auditory stimuli.

Importantly, although the alternative methods mentioned by the reviewer can capture non-linear representations as well, they lack sufficiently detailed information about the nature of the neural response itself, whereas TRF provide insight into the spatio-temporal features of the neural response and are thus is physiologically interpretable.

The functional relevance of TRFs has also been validated in the domain of hearing and speech processing, with correlations demonstrated between neural speech-tracking metrics and speech intelligibility, for example in children with dyslexia or in those with hearing impairment (Keshavarzi et al., 2022; Van Hirtum et al., 2023; Xiu et al., 2022), and of course in the domain of selective attention (Ding et al., 2012; Fiedler et al., 2019; Kerlin et al., 2010; J. A. O'Sullivan et al., 2015; Zion Golumbic, Ding, et al., 2013, Kaufman &Zion Golumbic, 2023). For these reasons, we have chosen to stick with this approach in the current work, although we fully acknowledge that its linear nature is somewhat limiting. We now acknowledge this in the Discussion (p. 25).

Regarding the choice to use the broadband speech envelope: Several previous studies have shown that when performing speech-tracking analysis of scalp-recorded E/MEG data, similar results are obtained if using a bank of narrow-band responses or averaging them into a broadband response. This might not be the case for recordings with higher spatial resolution (e.g. invasive recordings) which can capture more fine-grained spectro-temporal responses, however for the current purposes the broadband envelope is sufficient.

Other work has focused on lateralised changes to the oscillatory cerebral signal (particularly in the alpha band) as a function of lateralised auditory attention (Wöstmann and colleagues, e.g. Wöstmann, Alavash &Obleser, 2019; Wöstmann, Lim &Obleser 2017). There is no mention of this research, which seems like an oversight when summarising the current state of the field, even if the TRF method is not directly comparable to time-frequency analysis.

We are aware of this work and did look at alpha power but did not find any interesting effects, nor did we see convincing lateralization (see Figure below). For this reason, we did not include this in the manuscript, however if the reviewer feels that this is pertinent, we are happy to add these null results to the paper.

Minor points and requests for clarification:

Specify the interpolation method applied, We used the built-in Fieldtrip toolbox FT_CHANNELREPAIR function which repairs bad channels in the data by replacing them with the plain average of all neighbors (see Perrin et al., 1989). Neighbors were defined as all channels within a distance of up to 0.15 cm from the bad channel. We have now added this detail to the manuscript (p.7). describe how cardiac artefacts were identified ICA components capturing cardiac artifacts were identified visually. These components show a typical rhythmically-recurring spikes that are reminiscent of AKG recordings, and often show a diagonal topography over the scalp. We have now clarified in the manuscript that identification was done through visual inspection (p.7).

Please clarify or homogenise: "Trials" and "Sentences" Perhaps the reviewer meant "segments" rather than "sentences"? We use the term "segment" to refer a portion of the speech-stimulus, and the term "trial" to refer to the data collected while participants heard a particular "segment" of the lecture.

We have now clarified that (p.7): "The clean data was cut into trials, corresponding to portions of the experiment in which a single segment of the lecture was presented." Please provide the reference for "Liberman's cochlear frequency map" We have added the reference to Liberman (1982) to the manuscript Provide more detail on the calculation of Bayes factors - report the priors in sufficient detail to allow future replication of the analyses, even if the defaults of the software should change, or in other software.

The Bayesian ANOVA was conducted using JASP (version 0.16.3) with default settings. Fixed effects were modeled using a Cauchy prior distribution with a scale parameter of r=0.5. Random effects were modeled using a Cauchy prior with a scale parameter of r=1, and covariates were modeled using a Cauchy prior with a scale parameter of r=0.354. A uniform prior was applied to the model space, assigning equal prior probabilities to all models. The principle of marginality was respected, ensuring that all lower-order effects were included in models with higher-order interactions. We have now added these details to the manuscript (p.8).
