# Supplementary figures and images for: Neural Speech Tracking during Selective Attention: A Spatially Realistic Audiovisual Study
Source: eNeuro. 2025 Jun 18;12(6):ENEURO.0132-24.2025. doi: 10.1523/ENEURO.0132-24.2025 (PMC12203769; doi:10.1523/ENEURO.0132-24.2025)

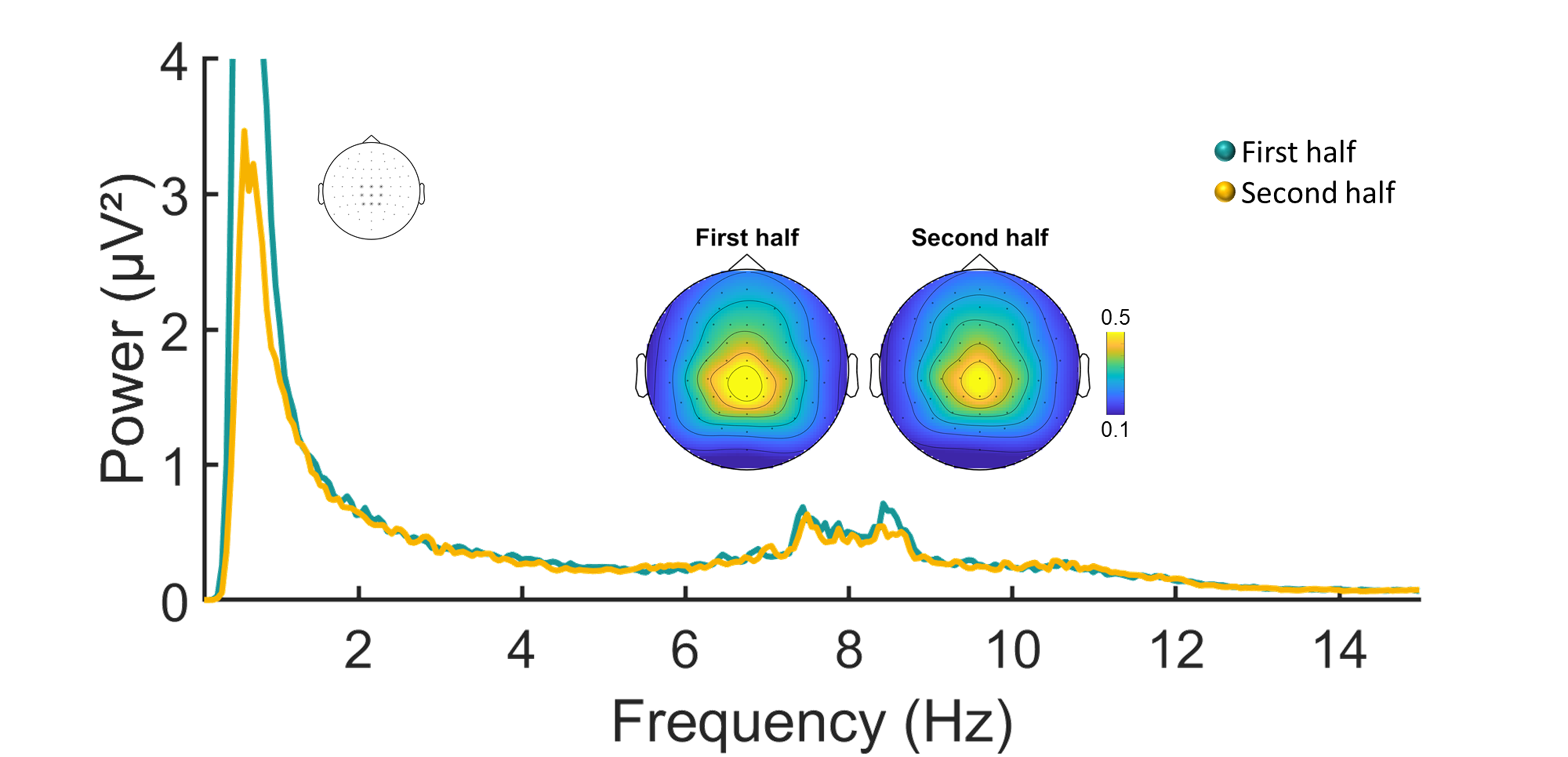

Supplement: Figure 4-1 — Group-level Spectral analysis. Comparison of the power spectrum of the EEG between the first and second half of the experiment. We calculated the spectral power density using a multitaper fast-fourier transform (FFT; as implemented in Fieldtrip), separately for the data from each half of the experiment. Shown in the figure is the power spectrum averaged over 9 centro-parietal electrodes (marked in the subplot on the top-left). A clear peak is seen in the low alpha-range (7-9 Hz) in both halves of the experiment, which was maximal at centro-parietal electrodes (shown in the topographies). However, a paired t-test revealed no significant difference between alpha power in the first vs. second half of the experiment [t(22) = 1.25, p = 0.22], which might have been expected as an index of fatigue or reduced attention over time (e.g. Yu et al., 2021). Download Figure 4-1, TIF file. [file eneuro-12-ENEURO.0132-24.2025-s001.tif]

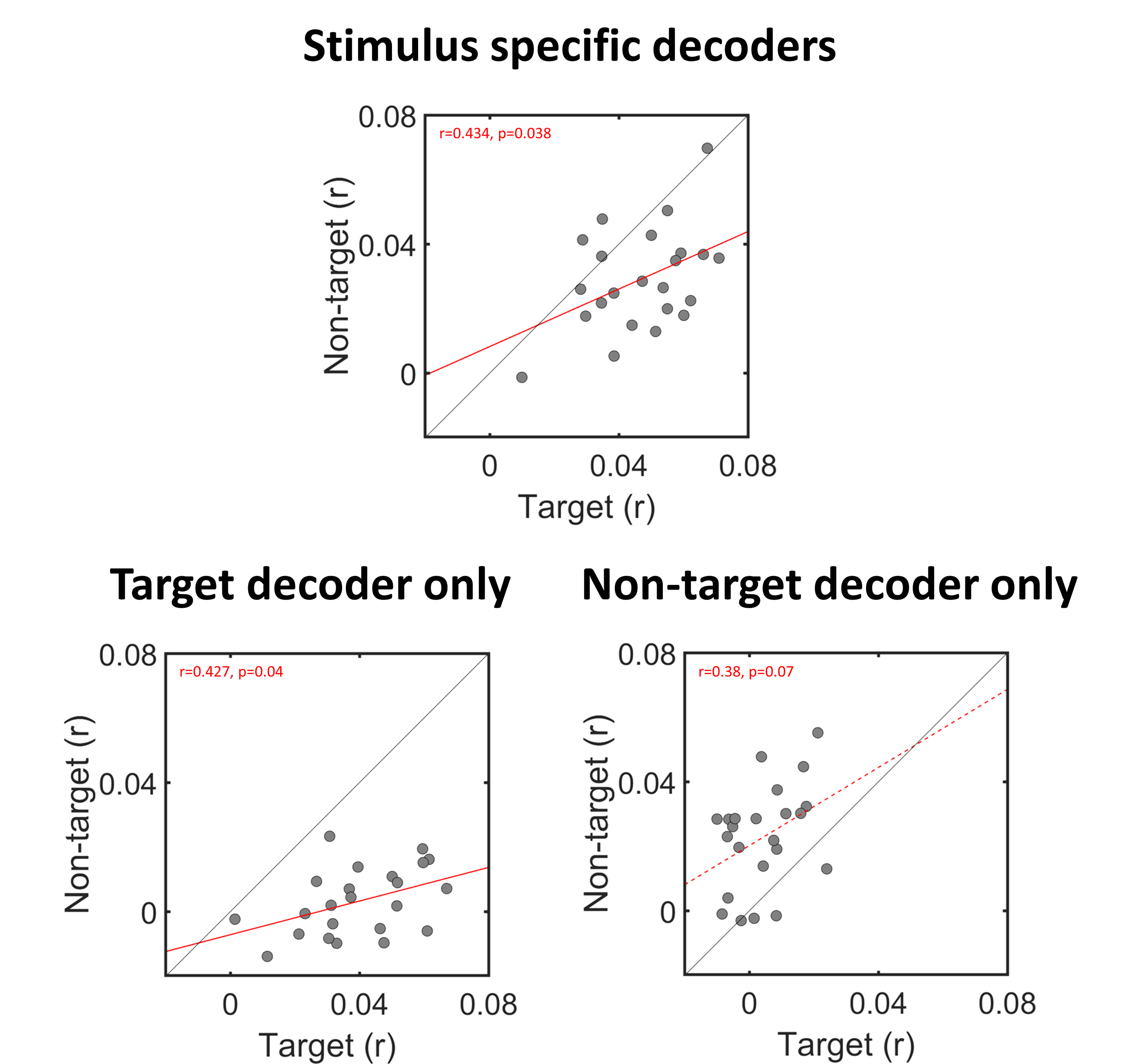

Supplement: Figure 6-1 — Comparison of decoder-testing approaches. Here we compare two approaches for testing the performance of decoders trained on EEG data to reconstruct the envelope of concurrently presented speech. Top: The approach used and reported in the current study, in which two Stimulus specific decoders were trained using a multivariate approach to reconstruct the envelopes of target and non-target speech presented concurrently. The scatter-plot shows reconstruction accuracies achieved for both decoders across all participants, when tested on left-out data of the same type (i.e., how well the target decoder can reconstruct left-out target speech, and how well the non-target decoder can reconstruct left-out non-target speech). The gray line reflects the diagonal, and the red line represents the linear regression fit between the two variables which was statistically significant [data is the same as in Figure 6A]. Bottom: Re-analysis of the same data using the auditory attention-decoding (AAD) approach, in which a decoder is trained only on one stimulus (e.g., on target speech), and is then tested on left-out data of the same stimulus (target) and of the other stimulus (non-target), and the two results are compared for classification purposes. The left panel shows a scatter-plot showing how well a decoder trained on target speech can reconstruct left-out target speech vs. how well it can reconstruct left-out non-target speech, across all participants. The left panel shows the same for a decoder trained on non-target speech. The gray line reflects the diagonal, and the red line represents the linear regression fit between the two variables (dashed line indicates a marginally significant regression). In this analysis, almost all dots fall either below or above the diagonal, clearly showing between reconstruction performance when a decoder is tested on data of the same type that it was trained on. This is in line with multiple studies, that propose using this approach for practical a [file eneuro-12-ENEURO.0132-24.2025-s002.tif]
